# Impact of low-carbohydrate diet on health status: an umbrella review

**DOI:** 10.3389/fnut.2024.1321198

**Published:** 2024-09-25

**Authors:** Sarah Alkhunein, Rehab Alawad, Omar Alhumaidan, Bushra Fatani, Abeer Alolayan, Tarfah Alfelyeh, Shihana Alakeel, Yara Almuhtadi

**Affiliations:** ^1^National Nutrition Committee (NNC), Saudi Food and Drug Authority (SFDA), Riyadh, Saudi Arabia; ^2^Department of Community Health Sciences, College of Applied Medical Sciences, King Saud University, Riyadh, Saudi Arabia

**Keywords:** low-carbohydrate diet, ketogenic diet, nutrition, health, umbrella review

## Abstract

**Introduction:**

The prevalence of diet-related non-communicable diseases has increased. A low-carbohydrate diet (LCDs) is one of the most popular interventions. Several systematic reviews and meta-analyses of randomised clinical trials (RCTs) and non-RCTs have linked LCDs to the management of obesity, diabetes, cardiovascular disease, epilepsy, and cancer. However, there has been limited appraisal of the strength and quality of this evidence.

**Objective:**

To systematically appraise existing meta-analyses and systematic reviews of RCTs and non-RCTs on the effects of LCDs on different health conditions. To understand their potential efficacy, we summarised the studies' findings and assessed the strength of the evidence.

**Methods:**

A search was conducted using the PubMed database from inception to October 2021 for systematic reviews and meta-analyses of RCTs and non-RCTs investigating the association between LCDs and multiple health outcomes in humans. The Academy of Nutrition and Dietetics Quality Criteria was used for the quality assessment. In addition, the evolution of heterogeneity, strength of the included studies, and effect sizes were extracted from each systematic review and meta-analysis.

**Results:**

Ten systematic reviews and meta-analyses were included. Of the included reviews, 70% were of positive quality, 30% were neutral, and none were negative. The majority of the studies included strength in each systematic review, and the meta-analyses were of low to medium strength. The existing literature indicates that LCDs may help promote weight reduction in adults who are obese or overweight. This conclusion is supported by the findings of studies included in the analysis, which were of low to moderate strength. Furthermore, compelling data indicates a significant association between low-carbohydrate diets (LCDs) and a reduction in haemoglobin A1c levels among those diagnosed with type 2 diabetes mellitus. In contrast, there was a lack of evidence of this correlation in type 1 diabetes mellitus patients or those with cardiovascular diseases. Additionally, there was limited evidence regarding the effectiveness of LCDs in epilepsy and adult cancer patients.

**Conclusion:**

This review thoroughly examines the current body of information on how LCDs affect various health outcomes. Studies have presented evidence to support the idea that incorporating LCDs can positively influence weight management and HbA1c levels. However, there is a lack of information regarding the association between LCDs and individuals with Type 1 diabetes mellitus and cardiovascular diseases. Additionally, there is limited empirical evidence to substantiate the effectiveness of LCDs in the treatment of epilepsy and adult cancer patients. The long-term effects of LCDs on mortality and other chronic diseases that account for different carbohydrate subtypes is unclear. Further longitudinal cohort studies are required to reach definitive conclusions.

## 1 Introduction

Dietary interventions may affect Non-Communicable Diseases (NCDs), which are considered a global public health challenge (1). NCDs are the leading cause of death worldwide, killing over 41 million people annually and accounting for 74 % of all deaths (2, 3). Although the aetiology of NCDs remains unclear, unhealthy diet is emerging as a major modifiable risk factor (4, 5). Thus, there has been an increased interest in the roles of diets and different dietary macronutrient distribution patterns in preventing and managing the severity of NCDs.

Dietary carbohydrates (CHO) are essential macronutrients that provide the body with energy and support its physiological functions (6). Consequently, the recommended intake differs according to age, body weight, physical activity and health conditions. However, the Acceptable Macronutrient Distribution Range for healthy adults is 45%−65% of the total calorie requirement (7). The various low carbohydrate dietary approaches all restrict the total consumption of carbohydrates to some degree, yet there is a lack of definitive consensus regarding the exact parameters that define low-carbohydrate diets (LCDs). Nonetheless, most LCDs involve decreasing carbohydrate intake to < 45% of an individual's total caloric intake.

The different terms used for LCDs often imply differences in the distribution of macronutrients. For example, the Atkins, South Beach, and Zone diets are characterised by < 40% CHO, ~30% protein, and 30%−55% fat (8, 9). Additionally, the ketogenic diet (KD) was the most restrictive diet among LCDs, which includes 5%−10% CHO, about 10% protein, and replaces the remaining with dietary fat (9).

Several epidemiological studies have shown that high CHO intake is potentially associated with an increased risk of many diseases such as cancer, heart disease, diabetes, and metabolic syndrome (10–14). However, numerous systematic and meta-analysis reviews have shown that subsequent LCDs may lead to some potential improvements in metabolic risk factors, such as haemoglobin A1C (HbA1c) and lipid profile and may help in weight loss (8, 15–22). Nonetheless, studies on the strength and quality of this evidence are limited (8, 15–22). To date, two recent umbrella reviews have been published 2021-2023 have assessed the quality of evidence (23, 24). The first review included 17 meta-analyses of randomised control trials (RCTs) that assessed the association between KD and health outcomes. They found that the KD reduced triglyceride (TG) by MD, −18.36 mg/dl after 3 months and by MD, −24.10 mg/dl after 12 months compared with regular diet and increased low-density lipoprotein (LDL) by MD, 6.35 mg/dl for 12 months compared with regular diet in adults (23). In contrast, the KD decreased seizure frequency in children and adolescents with epilepsy by RR, 5.11 for 3–16 months when compared with regular diet (23).

The second review included 43 meta-analyses of observational studies addressing the association between CHO and 23 health outcomes categorised into five primary domains: (1) 11 types of cancer, (2) all-cause and cause-specific mortality, (3) metabolic diseases including T2DM and metabolic syndrome, (4) digestive system conditions including ulcerative colitis, Crohn's disease, and inflammatory bowel disorders, and (5) other outcomes comprising coronary heart disease, stroke, Parkinson's disease, and bone fracture (24). Based on a rigorous assessment of the quality of evidence, the study found high-quality evidence associating higher CHO intake with an increased incidence of metabolic syndrome, and potential link of high mortality, as well as a decreased likelihood of developing oesophageal cancer. However, the evidence regarding the relationship between carbohydrate intake and other health outcomes appears to be inconclusive or lacking.

Previous efforts of published umbrella reviews have focused on assessing either one type of LCDs (KD) or the included meta-analyses of observational studies. Hence, the present umbrella review aimed to systematically appraise existing meta-analyses and systematic reviews of RCTs and non RCTs on the effects of LCDs on multiple health conditions to understand their potential efficacy, summaries the studies' findings, and assess the strength of evidence to provide a comprehensive vision.

## 2 Materials and methods

### 2.1 Design

This umbrella review was conducted to synthesis and investigate the quality of all existing published systematic reviews and meta-analyses of RCTs and non-RCTs to assess the association between LCDs and diverse health conditions. This review was performed according to the 2020 PRISMA Statement and all items of the PRISMA checklist were completed (25).

### 2.2 Search strategy

Two independent investigators conducted a search using the PubMed database from its inception to October 2021 to identify systematic reviews and meta-analyses of RCTs and non-RCTs that assessed evidence regarding the effects of LCDs on health. The keywords used in this search strategy were: “carbohydrates,” “carbohydrate,” “carb,” “fat,” “proteins,” “proteinous,” “protein,” “ketogenic,” “keto,” and “atkins.” Search philtres were used to identify systematic reviews, meta- analyses, and full- text articles. All titles and abstracts were screened to remove duplicates and select potentially eligible articles. Discrepancies were resolved through discussion with a third investigator.

### 2.3 Study eligibility criteria

Articles were eligible for inclusion if they were systematic reviews and meta-analyses of RCTs and non RCTs including human studies across all age groups, and articles that only aimed to investigate the associations between LCDs and health outcomes, with a restriction that the amount of CHO did not exceed 45% of the total daily caloric intake, were included. Articles meeting the following criteria were excluded: systematic reviews and meta-analyses based on animal studies, experiments with designs other than RCTs or non RCTs, studies that included diets with more than 45% CHO, and articles published in language other than English.

### 2.4 Data extraction

The research team developed a data extraction form. The data extracted from each systematic review and meta-analysis included the first author's name, publication year, study design, number of studies included in each systematic review and meta-analysis, study population, intervention, comparison group, duration, parameters of interest, evolution of heterogeneity (I^2^%), and quality of the studies included in each systematic review and meta-analysis. Data were independently extracted by two investigators. Disagreements were resolved through discussion with a third investigator.

### 2.5 Assessment of the quality of systematic reviews and meta-analyses

The quality of systematic reviews and meta-analyses was evaluated using the Quality Criteria Checklist created specifically for primary research articles by the Academy of Nutrition and Dietetics (26) and graded into three categories: “(1) Positive: indicates that the review has clearly addressed issues of inclusion/exclusion, bias, generalizability, and data collection and analysis. (2) Negative: indicates that these issues have not been adequately addressed. (3) Neutral: indicates that the review is neither exceptionally strong nor weak.” Quality assessment was independently performed by four investigators. The decision was made when three of the four investigators agreed, and all discrepancies were resolved through a discussion with a fifth investigator. To ensure the accuracy of the evaluation, we shared the quality assessment with five Ph.D. nutrition experts. The evaluation was subsequently revised based on the expert feedback and comments.

### 2.6 Data analysis

The calculation of the effect size was measured in current review by Cohen's *d*. The effect size, denoted as *d*, is calculated manually using the following equation (27): *d* = (M1 – M2)/spooled. Here, M1 represents the mean of group 1, M2 represents the mean of group 2, and spooled refers to the pooled standard deviations for the two groups. It is crucial to note that the interpretation of the effect size depends on its value. If d falls within the range of ≥ 0.2 ≤ 0.499, it is considered a “small” effect. A value of ≥ 0.5 ≤ 0.799 indicates a “medium” effect, while a value of ≥0.8 suggests a “large” effect (28). We assessed heterogeneity using the *I*^2^ statistics, considering *I*^2^% ≥50% as indicative of high heterogeneity (29).

## 3 Results

### 3.1 Search results

**Figure 1:**
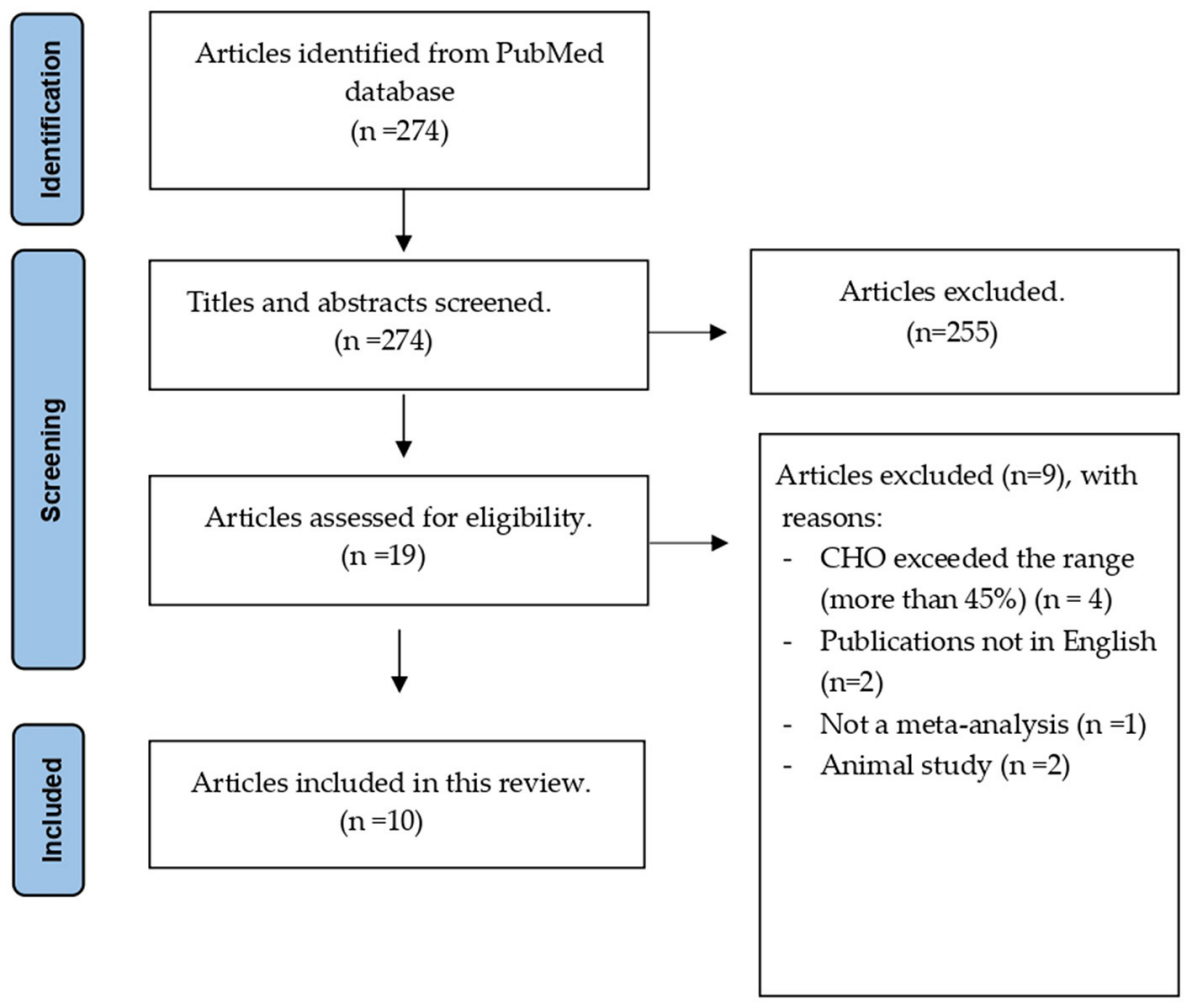
Articles selection flowchart.

A total of 274 articles were initially identified, and 255 were excluded after title and abstract screening. Nine of the 19 articles were excluded after applying the inclusion criteria. Finally, 10 eligible articles were included, as shown in Figure 1. A list of the excluded articles is presented in Supplementary material.

### 3.2 Characteristic of the included systematic reviews and meta-analyses

Among eligible systematic reviews and meta-analyses, different outcomes associated with five health conditions were examined: obesity, cardiovascular disease, diabetes, cancer, and epilepsy.

The characteristics of the included reviews are summarised in Table 1. The average number of studies included in each systematic review and meta-analysis was 31 (range: 6–121), and the average number of participants was 4,067 (range: 222–21,942). The participants in all the included reviews were adults, except for one review that included both children and adults (30). Most participants in the included studies were obese or overweight (80%) (8, 17–22, 31), followed by those with T2DM (40%) (17, 19, 22, 31). The most frequently measured health indicators in the included studies were weight (body weight (BW), waist circumference (WC), and body mass index), blood sugar levels [fasting blood glucose (FBG) and haemoglobin A1C (HbA1c)], and lipid profiles.

**Table 1:** General characteristics of included systematic reviews and meta-analyses.

**Study number**	**References**	**Study design of studies included in each systematic review and meta-analyses**	**Number of studies included in each systematic review and meta-analyses**	**Population**	**Intervention**	**Comparison group**	**Duration**	**Parameters of interest**
1	Yuan et al. (31)	7 RCTs and 6 non-RCTs	13	Obese or overweight adults with T2DM	KD	Pre-KD	1–56 weeks	TC
								TG
								HDL
								LDL
								BW
								HbA1C
								FBG
2	Choi et al. (17)	RCTs	14	Obese or overweight adults with or without T2DM	KD	LFDs	2 h to 12 months	HDL
								TG
								BW
								HbA1C
3	Sackner-Bernstein et al. (18)	RCTs	17	Obese or overweight adults	LCDs	LFDs	2–24 months	BW
4	Ge et al. (8)	RCTs	121	Obese or overweight adults	LCDs	Moderate macronutrient dietary patterns (CHO: **>**40**%** of TEI)	6 months	BW
5	Schwingshackl et al. (19)	RCTs	56	Obese or overweight adults	LCKDs + exercise	Usual diet + exercise	4–24 weeks	HbA1C
6	Lee and Lee (20)	RCTs	7	Obese or overweight adults	LCKDs + exercise	Usual diet + exercise	4–24 weeks	WCTG
7	Chawla et al. (21)	RCTs	38	Obese adults	LCDs	LFDs	1–24 months	HDL
								TG
8	Yang et al. (32)	RCTs	6	Adults with cancer	KD	Non-KD	4-24 weeks	PSA—Tumour marker
9	Martin-McGill et al. (30)	RCTs	13	Children and adults with epilepsy	KD	Usual care	2–16 months	- Seizure freedom - 50% or greater reduction in seizure frequency
10	Hu et al. (22)	RCTs	23	Adults with metabolic risk	LCDs	LFDs	6-24months	HDL TG

T2DM, Type 2 diabetes mellitus; KD, ketogenic diet; CHO, carbohydrate; TC, total cholesterol; TG, triglyceride; HDL, high-density lipoprotein; LDL, low-density lipoprotein; BW, body Weight; HBA1C, Haemoglobin A1C; FBG, fasting blood glucose; LFDs, low-fat diets; LCDs, low carbohydrate diet; LCKDs, low-carbohydrate ketogenic diets; WC, Waist Circumference; PSA, Prostate-Specific Antigen; TEI, Total Energy intake; RCTs, randomised clinical trials.

### 3.3 Quality and strength of evidence

Quality of evidence indicates the extent to which the article has clearly addressed the issues of inclusion/exclusion, bias, generalizability, and data collection and analysis. The quality of the included systematic reviews and meta-analyses was assessed using the Academy of Nutrition and Dietetics Quality Criteria Checklist, which categorised the quality of evidence into three categories (positive, negative, and neutral) (26). Seven of the 10 eligible reviews (70%) were positive (8, 17, 19–21, 30, 32), three of the 10 eligible reviews were neutral (30%) (18, 22, 31), and none of the included reviews were negative, as shown in Table 2. Based on the findings, the strengths of the included studies as reported by the included systematic reviews and meta-analyses were low to moderate in four of 10 eligible reviews (40%) (8, 17, 19, 21); low to very low in two reviews (20%) (20, 30); and moderate to high strength evidence in one review. Grading information was unavailable for three of 10 included reviews (30%) (18, 22, 31) as shown in Table 2.

**Table 2:** Heterogeneity, strength of the included studies, quality of the included reviews, and effect size.

**References**	**Parameters of interest**	**Evolution of heterogeneity (*I*^2^%)**	**The strength of the studies included in each systematic review and meta-analyses**	**The quality of the included systematic reviews and meta-analyses**	**Effect size**
Yuan et al. (31)	TC	75%	N\A	Neutral	Medium effect
	TG	67%			Large effect
	HDL	78%			Small effect
	LDL	71%			Small effect
	BW	92%			Large effect
	HbA1C	68%			Medium effect
	FBG	71%			Large effect
Choi et al. (17)	HDL	10%	Low to moderate	Positive	Medium effect
	TG	59%			Medium effect
	BW	78%			Medium effect
	HbA1C	23%			Medium effect
Sackner-Bernstein et al. (18)	BW	N\A	N\A	Neutral	Large effect
Ge et al. (8)	BW	N/A	Low to moderate	Positive	Large effect
Schwingshackl et al. (19)	HbA1C	N\A	Low to moderate	Positive	Small effect
Lee and Lee (20)	WC	0%	Low	Positive	Large effect
TG	0%			Medium effect
Chawla et al. (21)	HDL	N/A	Low to moderate	Positive	Small effect
TG	N\A			Small Effect
Yang et al. (32)	PSA—Tumour marker	78.3%	Moderate to high	Positive	Large Effect
Martin-McGill et al. (30)	Seizure freedom	0%	Low to very low	Positive	The association not statistically significant
	50% or greater reduction in seizure frequency	0%			The association not statistically significant
Hu et al. (22)	HDL	78.6%	N\A	Neutral	Large effect
	TG	55.6%			Large effect

TC, total cholesterol; TG, triglyceride; HDL, high-density lipoprotein; LDL, low-density lipoprotein; BW, body Weight; HBA1C, haemoglobin A1C; FBG, fasting blood glucose; WC, waist circumference; PSA, prostate-specific antigen; NA, not available.

### 3.4 Summary effect size

**Figure 2:**
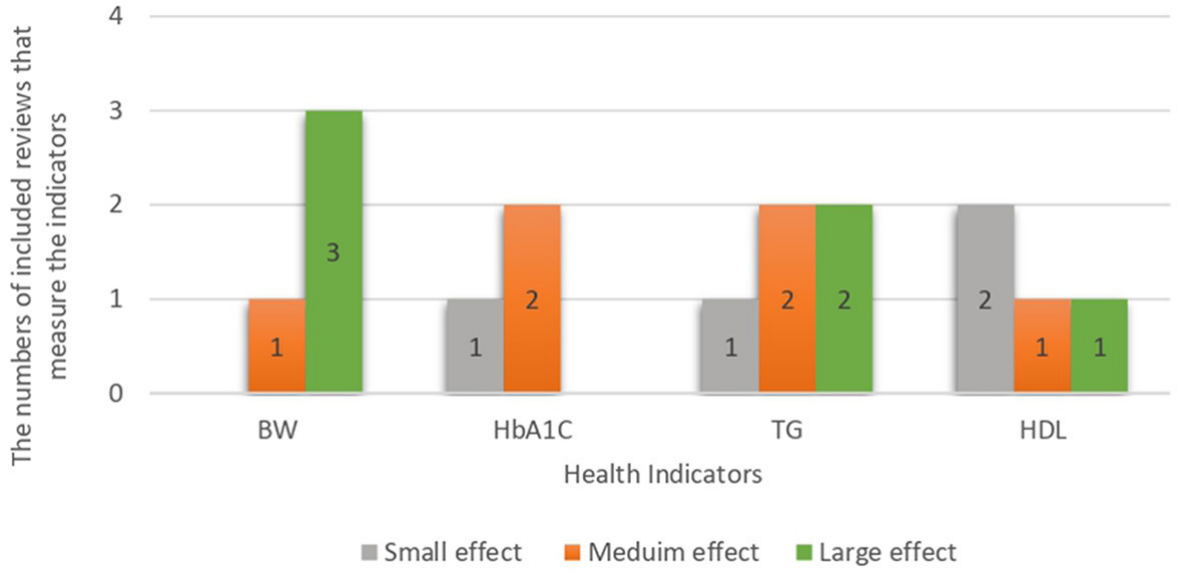
Effect size of most common health indicators in the studies included regardless of the disease.

The results showed that TG, HbA1c, high-density lipoprotein (HDL), and BW were significantly associated with LCD in most of the included systematic reviews and meta-analyses. Figure 2 summarises the effect sizes of all included reviews with similar health indicators for comparison. The findings revealed that most of the reviews reported statistical significance associations between HDL and LCD; however, the effect size was small. In terms of blood glucose indicators, HbA1c was the most frequently reported statistically significant indicator with a medium effect size. Among weight measurement indicators, the association between BW and LCD was considered to have a large effect size.

### 3.5 Heterogeneity between studies

There was high heterogeneity in most health indicators in the included studies (52%) and low heterogeneity in six health indicators (26%). In addition, 22% were not available. Systematic reviews and meta-analyses with high heterogeneity included health indicators such as TC, HbA1c, and FBG, as shown in Table 2.

### 3.6 Weight reduction outcomes

Five of the included reviews measured the indicators of weight gain (BW and WC) in obesity or overweight participants (8, 18, 20) and in participants with T2DM (17, 31). Among the included reviews, BW was the most frequently measured weight indicator. LCDs were linked to a BW decrease in individuals who were obese or overweight (8, 18), as well as those who had T2DM in adults (17, 31). However, only two of the included reviews had a strength rating of low to moderate, and their quality was positive, demonstrating that LCDs were associated with BW reduction with the mean difference of BW (−1 to 2, −3.81, and −7.78 kg in diabetes patients) (8, 17). Moreover, the effect size were medium to large Conversely, the reduction of WC by −0.74 cm was observed in one review with large effect size and *P* = 0.01; however, the strength of evidence was low (20). Furthermore, the findings suggest that weight reduction is achievable if proper calories are estimated for weight loss regardless of the carbohydrate composition of the diet.

### 3.7 Cardiovascular diseases outcomes

The types of LCDs had different effects on risk factors associated with CVDs (total cholesterol, TG, HDL, and LDL) in different populations (17, 20–22, 31). However, there is a lack of studies that directly measure the incidence of CVDs or that were conducted on CVD patients. LCDs have been associated with decreased TG and increased HDL levels which are considered to reduce the risk of CVD. Different effect sizes between studies for TG and HDL as shown in Figure 2. These results were obtained in obese and overweight participants, with or without T2DM, and in participants with metabolic syndrome (17, 20–22, 31). Nevertheless, these results are supported by a low-to-moderate level of evidence. A limited review of Neutral qualities found that LCD was associated with decreased LDL with small effect size and TC levels with medium effect size (31).

### 3.8 Diabetes outcomes

Three of the included reviews compared the efficacy of LCDs and other diets on HbA1C percentage. The results from these reviews showed that LCDs resulted in a significant reduction in the percentage of HbA1c compared to other diets (−0.82 to −0.47%, −0.5% to −0.42%, and 1.07%) in T2DM patients with medium effect size (17, 31), and small effect size (19). Furthermore, data from one review with neutral quality showed that a who consumed the control diet (−1.29 mmol/L) with large effect size (31).

### 3.9 Cancer outcomes

Overall, there is limited evidence regarding the safety and effectiveness of LCDs in cancer patients. One included review with positive quality, moderate-to-high strength of included studies, and a large effect size found a statistically significant association between LCDs and one of the crucial tumour markers for prostate cancer (PSA); *P* = 0.03 in prostate cancer (32).

### 3.10 Epilepsy outcomes

A Cochrane review suggested that high levels of ketones in the blood caused by KD may reduce the frequency of epileptic seizures in children with drug-resistant epilepsy (30). However, it should be noted that some children experience frequent adverse effects of LCDs, such as constipation, vomiting, nausea, and diarrhoea (30). In adults, the current review showed that there are limited systematic reviews and meta-analyses of RCTs and non-RCTs on the effectiveness of LCDs in epilepsy patients, and the results of the evidence are uncertain (30).

## 4 Discussion

### 4.1 Principal findings and possible mechanisms

This umbrella review provides an overview and appraisal of 10 systematic reviews and meta-analyses o RCTs and non RCTs on the effects of LCDs on different outcomes associated with five health conditions obesity, CVDs, T2DM, cancer, and epilepsy. Seventy percent of the included reviews were of positive quality (seven reviews), 30% were neutral (three reviews), and none were negative.

Although most reviews were of positive quality, the strengths of the studies included in each systematic review and meta-analysis ranged from low to medium. The current review includes a systematic and meta-analysis that evaluated prospective cohorts and RCTs studies. These studies included participants from several geographic locations, as well as ethnic and cultural diversity, to minimise variations that could potentially bias the findings. Some of the included meta-analyses contained duplicate articles (primary studies), due to the use of similar keywords during the search phase. In addition, most of the meta-analyses were published between 2020 and 2021. However, this review design focused on summarising the evidence, comparing the effect sizes across all investigated factors, and reporting the heterogeneity across the included studies in each meta-analysis. Pooling the data derived by the same studies, create forest plots, and other summing analyses may brought together to provide one estimate and cause overlapping by over/underestimation of some results, for that reason each meta-analysis review was assessed separately.

Multiple studies have identified various biological mechanisms that indicate significant metabolic and hormonal changes upon achieving high ketone body production or ketosis, in relation to the five health conditions focused in this review (33, 34). Ketone bodies produced by the liver serve as oxidative fuels and markers of carbohydrate deficiency. They may help conserve carbohydrates during periods of low carbohydrate availability by reducing insulin levels and anabolic processes and promoting the conversion of fatty acids from fat storage and diet into ketone bodies for energy (35, 36).

The utilisation of fats for ketone bodies production as energy has led some studies to suggest potential weight loss as a risk factor for the five health conditions on our review. This concept, known as the carbohydrate-insulin model of obesity, suggest that high-glycemic carbohydrates encourage fat storage and hunger, which can be mitigated through lifestyle changes, such as prolonged fasting, extended exercise, or a low-carbohydrate diet to lower insulin levels (37, 38). According to this model, low-carbohydrate diets may reduce the risk of obesity, CVDs, T2DM, and cancer by decreasing body weight and increasing fat utilisation by limiting carbohydrate intake. However, for epilepsy, there was no direct mechanism that suggests how weight loss in the carbohydrate-insulin model of obesity might help improve the outcomes of this condition.

According to the epidemiological study, a reduction of 5 kg/m^2^ of height can lead to an average of 27% decrease in the risk of CVDs and a 17% decrease in the risk of T2DM. Additionally, this reduction in body mass may decrease the risk of pancreatic cancer by 14%, colon cancer by 3%, breast cancer by 3%, uterine cancer by 52%, kidney cancer by 31%, bladder cancer by 23%, and liver cancer by 59% (39, 40). Consequently, the effectiveness and efficiency of weight loss resulting from LCD are crucial factors in achieving results that are comparable to those achieved from these epidemiological studies.

Although the expected physiology and mechanisms appeared promising, the evidence reviewed in this paper showed varying degrees of effectiveness when such diet was tested. There was evidence suggesting the effectiveness of LCDs on weight reduction in obesity or overweight adults, supported by the low to moderate strength of the included studies. Moreover, evidence strongly suggested a correlation between LCDs and HbA1c reduction in T2DM patients. In contrast, systematic reviews and meta-analyses of RCTs and non-RCTs that included T1DM patients or those with CVDs are lacking. In addition, the current review showed that there is limited evidence regarding the effectiveness of LCDs in epilepsy and adult cancer patients.

Epidemiological studies suggests that the harmful impact of such diets may be attributed to the significant concern of increased overall fat intake (41). The results indicate that an increase in calories derived from fats can lead to a rise in total serum cholesterol levels by ~0.02 mm/L. Additionally, a change of 1 mm/L in total cholesterol could potentially increase the risk of mortality from CVDs by 20%, as indicated by research (42). This might explain the lack of significant findings regarding CVDs on our review. However, it is important to note that the majority of the studies included in this review have not measured the change in cholesterol levels resulting from high fat intake and their subsequent association with CVDs risk outcomes. Instead, the primary focus has been on the direct dietary intake of LCDs in relation to CVDs. Therefore, further research in this area may be necessary to better understand the dose-response relationship, particularly with regards to this specific focus.

### 4.2 Comparison with other reviews and possible explanations

According to the European Food Safety Authority, individuals are recommended to consume carbohydrates within 45%−60% of their total energy intake (43). This dietary guideline effectively improves metabolic risk factors associated with chronic diseases when combined with reduced total fat and saturated fat consumption (44). Nevertheless, a precise consensus about the definition of LCDs has yet to be reached. Most LCDs typically involve reducing CHO intake to below 45% of the total caloric intake.

Two umbrella reviews have examined the impact of LCDs on various health outcomes. An umbrella summarises and evaluates 43 meta-analyses of observational studies investigating the association between CHO intake and health outcomes, including cancer, mortality, metabolic diseases, digestive system outcomes, and other outcomes (coronary heart disease, stroke, Parkinson's disease, and bone fracture). The findings of this review provided evidence in favour of the correlation between carbohydrate intake and metabolic syndrome. In addition, the researchers proposed that the evidence presented on the negative impact of CHO consumption on coronary heart disease and T2DM was lacking in strength. This finding is consistent with the results of our present review, which indicate that LCDs may enhance risk factors associated with cardiac illnesses and T2DM. Nevertheless, the findings presented in this study are supporting by a low to moderate degree quality of evidence. Additionally, a statistically significant correlation was seen between the use of LCDs and the proportion of HbA1c levels in individuals with T2DM (24).

A recent umbrella review was conducted to systematically identify and summarise relevant meta-analyses of RCTs on KD (23). The analysis included data from 17 meta-analyses, which consisted of a total of 68 RCTs (23). The primary objectives of the review were to identify the effects of KD on health outcomes and to assess the strength of the evidence supporting these effects (23). The study demonstrated positive correlations between KD and various cardiometabolic markers. Furthermore, it was observed that KD had inconsistent effects on TC and LDL levels, resulting in unfavourable outcomes. However, the authors considered 76% of the RCTs to be critically low quality (23). On the contrary, our findings demonstrated an inverse relationship between LCDs and CVD, as evidenced by reduced TG levels and elevated HDL levels. However, it should be noted that the findings presented in this study are supported by a level of evidence that ranges from low to moderate. Additionally, the review demonstrated positive results linked to the KD, including changes in BW, TG, HDL-C, and HbA1c levels. The discovery above aligns with the outcomes obtained from our research. Furthermore, high-quality evidence supports the notion that a KD can effectively reduce the frequency of seizures by 50% or more in children and adolescents (23). This finding is consistent with a large retrospective cohort study that compared KD with usual care, which aim to assess the safety, effectiveness, and retention rate of KD for paediatric with drug-resistant epilepsy found that after KD, the retention rate significantly increased over time by 82.0% at 3 months, 60.6% at 6 months, and 34.1% at 12 months. Additionally, the response rate improved dramatically over 3 months by 55.5%, 6 months by 43.2%, and 12 months by 31.5% (45).

When looking at the effect of other diets such as high-protein diet, the Mediterranean diet, and high fibre diet. We found that there were evidence supporting the improvement of health marks by following these diets. However, there were limited meta-analysis that have been compared the LCDs with different diets types other than control diets. Systematic review and meta-analysis of different dietary approaches to the management of type 2 diabetes was published in 2013, aimed to assess the effect of various diets including (low-carbohydrate, vegetarian, vegan, high-fibre, low glycemic index, Mediterranean, and high-protein diets) on glycemic control, lipids, and weight loss. The results showed that the LCDs, low glycemic, Mediterranean, and high-protein diets were effective in improving different indicators of cardiovascular risk in diabetic people (46).

### 4.3 Strength and limitations

To the best of our current understanding, this umbrella review represents a pioneering effort in offering a methodical and all-encompassing evaluation of published systematic reviews and meta-analyses of RCTs and non-RCTs that examine the impact of LCDs on various human health-related outcomes. This review can assist dietitians, nutritionists, and researchers in evaluating the comparative efficacy of LCDs on many health outcomes. Furthermore, we assessed the methodological rigour. We examined the magnitude of the effect size and heterogeneity among the included reviews to guide future research endeavours.

One potential constraint of this review is the need for a comparative analysis of various LCD kinds, which exhibit variations in the extent of carbohydrate, fat, and protein limits. This omission may have influenced the outcomes of the study. A further factor worthy of consideration pertains to the predominant focus of existing studies on quantifying immediate results, hence neglecting the possibility of secondary effects associated with LCDs that could be discerned using alternative research methodologies. Furthermore, similar to other literature reviews, umbrella reviews are susceptible to biases, particularly appraisal and selection biases.

In summary, this umbrella review comprehensively examines the available information on the effects of LCDs on various health-related outcomes. Several studies have provided data supporting the notion that the utilisation of LCDs can have a beneficial effect on weight management and HbA1c levels. Nevertheless, there is a scarcity of information about the correlation between this connexion in patients with T1DM and CVDs. Furthermore, there is limited empirical support for the effectiveness of LCDs in treating epilepsy and adult cancer patients. Moreover, it is recommended that future research endeavours prioritise investigating the enduring impacts of adhering to LCDs on mortality rates, the prevention and treatment of other chronic diseases, and the examination of how various sources of CHO influence this process.

## Data Availability

The original contributions presented in the study are included in the article/Supplementary material, further inquiries can be directed to the corresponding author.

## References

[1] Corvalán C Reyes M Garmendia ML Uauy R. Structural responses to the obesity and non-communicable diseases epidemic: update on the Chilean law of food labelling and advertising. Obes Rev. (2019) 20:367–374. 10.1111/obr.12802 30549191

[2] WHO [Internet]. (2023). Noncommunicable diseases. Available at: https://www.who.int/news-room/fact-sheets/detail/noncommunicable-diseases (accessed September 19, 2023).

[3] Tyrovolas S El Bcheraoui C Alghnam SA Alhabib KF Almadi MAH Al-Raddadi RM . The burden of disease in Saudi Arabia 1990–2017: results from the Global Burden of Disease Study 2017. Lancet Planet Health. (2020) 4:e195–208. 10.1016/S2542-5196(20)30075-9 32442495 PMC7653403

[4] Kau AL Ahern PP Griffin NW Goodman AL Gordon JI. Human nutrition, the gut microbiome and the immune system. Nature. (2011) 474:327–36. 10.1038/nature10213 21677749 PMC3298082

[5] Varkevisser RDM van Stralen MM Kroeze W Ket JCF Steenhuis IHM. Determinants of weight loss maintenance: a systematic review. Obes Rev. (2019) 20:171–211. 10.1111/obr.12772 30324651 PMC7416131

[6] Cummings JH Stephen AM. Carbohydrate terminology and classification. Eur J Clin Nutr. (2007) 61:S5–18. 10.1038/sj.ejcn.1602936 17992187

[7] Trumbo P Schlicker S Yates AA Poos M. Dietary reference intakes for energy, carbohydrate, fiber, fat, fatty acids, cholesterol, protein and amino acids. J Am Diet Assoc. (2002) 102:1621–30. 10.1016/S0002-8223(02)90346-9 12449285

[8] Ge L Sadeghirad B Ball GDC Da Costa BR Hitchcock CL Svendrovski A . Comparison of dietary macronutrient patterns of 14 popular named dietary programmes for weight and cardiovascular risk factor reduction in adults: systematic review and network meta-analysis of randomised trials. BMJ. (2020) 369. 10.1136/bmj.m696 32238384 PMC7190064

[9] Bough KJ Rho JM. Anticonvulsant mechanisms of the ketogenic diet. Epilepsia. (2007) 48:43–58. 10.1111/j.1528-1167.2007.00915.x 17241207

[10] Sadeghi A Sadeghian M Nasiri M Rahmani J Khodadost M Pirouzi A . Carbohydrate quantity and quality affect the risk of endometrial cancer: a systematic review and dose-response meta-analysis. Clin Nutr. (2020) 39:1681–9. 10.1016/j.clnu.2019.08.001 31477367

[11] Huang J Pan G Jiang H Li W Dong J Zhang H . A meta-analysis between dietary carbohydrate intake and colorectal cancer risk: evidence from 17 observational studies. Biosci Rep. (2017) 37:BSR20160553. 10.1042/BSR20160553 28298476 PMC5469332

[12] Livesey G Livesey H. Coronary heart disease and dietary carbohydrate, glycemic index, and glycemic load: dose-response meta-analyses of prospective cohort studies. Mayo Clin Proc Innov Qual Outcomes. (2019) 3:52–69. 10.1016/j.mayocpiqo.2018.12.007 30899909 PMC6410335

[13] Alhazmi A Stojanovski E McEvoy M Garg ML. Macronutrient intakes and development of type 2 diabetes: a systematic review and meta-analysis of cohort studies. J Am Coll Nutr. (2012) 31:243–58. 10.1080/07315724.2012.10720425 23378452

[14] Liu YS Wu QJ Xia Y Zhang JY Jiang YT Chang Q . Carbohydrate intake and risk of metabolic syndrome: a dose–response meta-analysis of observational studies. Nutr Metab Cardiovasc Dis. (2019) 29. 10.1016/j.numecd.2019.09.003 31653521

[15] Luo W Zhang J Xu D Zhou Y Qu Z Yang Q . Low carbohydrate ketogenic diets reduce cardiovascular risk factor levels in obese or overweight patients with T2DM: a meta-analysis of randomized controlled trials. Front Nutr. (2022) 9:3101. 10.3389/fnut.2022.1092031 36583214 PMC9792675

[16] López-Espinoza MÁ Chacón-Moscoso S Sanduvete-Chaves S Ortega-Maureira MJ Barrientos-Bravo T. Effect of a ketogenic diet on the nutritional parameters of obese patients: a systematic review and meta-analysis. Nutrients. (2021) 13:2946. 10.3390/nu13092946 34578824 PMC8467306

[17] Choi YJ Jeon SM Shin S. Impact of a ketogenic diet on metabolic parameters in patients with obesity or overweight and with or without type 2 diabetes: a meta-analysis of randomized controlled trials. Nutrients. (2020) 12:2005. 10.3390/nu12072005 32640608 PMC7400909

[18] Sackner-Bernstein J Kanter D Kaul S. Dietary intervention for overweight and obese adults: comparison of low- carbohydrate and low-fat diets. a meta- analysis. PLoS ONE. (2015) 10:e0139817. 10.1371/journal.pone.0139817 26485706 PMC4618935

[19] Schwingshackl L Chaimani A Hoffmann G Schwedhelm C Boeing H. A network meta-analysis on the comparative efficacy of different dietary approaches on glycaemic control in patients with type 2 diabetes mellitus. Eur J Epidemiol. (2018) 33:157–70. 10.1007/s10654-017-0352-x 29302846 PMC5871653

[20] Lee HS Lee J. Effects of combined exercise and low carbohydrate ketogenic diet interventions on waist circumference and triglycerides in overweight and obese individuals: a systematic review and meta-analysis. Int J Environ Res Public Health. (2021) 18:828. 10.3390/ijerph18020828 33478022 PMC7835865

[21] Chawla S Silva FT Medeiros SA Mekary RA Radenkovic D. The effect of low-fat and low-carbohydrate diets on weight loss and lipid levels: a systematic review and meta-analysis. Nutrients. (2020) 12:3774. 10.3390/nu12123774 33317019 PMC7763365

[22] Hu T Mills KT Yao L Demanelis K Eloustaz M Yancy WS . Effects of low-carbohydrate diets versus low-fat diets on metabolic risk factors: A meta-analysis of randomized controlled clinical trials. Am J Epidemiol. (2012) 176. 10.1093/aje/kws264 23035144 PMC3530364

[23] Patikorn C Saidoung P Pham T Phisalprapa P Lee YY Varady KA . Effects of ketogenic diet on health outcomes: an umbrella review of meta-analyses of randomized clinical trials. BMC Med. (2023) 21:1–12. 10.1186/s12916-023-02874-y 37231411 PMC10210275

[24] Liu YS Wu QJ Lv JL Jiang YT Sun H Xia Y . Dietary carbohydrate and diverse health outcomes: umbrella review of 30 systematic reviews and meta-analyses of 281 observational studies. Front Nutr. (2021) 8:670411. 10.3389/fnut.2021.670411 33996880 PMC8116488

[25] PRISMA. PRISMA Checklist. (2020). Available at: http://prisma-statement.org/prismastatement/checklist.aspx?AspxAutoDetectCookieSupport=1 (accessed September 20, 2023).

[26] Handu D Moloney L Wolfram T Ziegler P Acosta A Steiber A. Academy of nutrition and dietetics methodology for conducting systematic reviews for the evidence analysis library. J Acad Nutr Diet. (2016) 116:311–8. 10.1016/j.jand.2015.11.008 26822985

[27] Statistics how to. Cohen's D: definition, Examples, Formulas - Statistics How To. Available at: https://www.statisticshowto.com/probability-and-statistics/statistics-definitions/cohens-d/ (accessed 2024 July 3).

[28] Cohen J. A power primer. Psychol Bull. (1992) 112:155–9. 10.1037//0033-2909.112.1.155 19565683

[29] Ioannidis JPA Patsopoulos NA Evangelou E. Uncertainty in heterogeneity estimates in meta-analyses. BMJ. (2007) 335:914–6. 10.1136/bmj.39343.408449.80 17974687 PMC2048840

[30] Martin-McGill KJ Bresnahan R Levy RG Cooper PN. Ketogenic diets for drug-resistant epilepsy. Cochrane Database Syst Rev. (2020) 6:CD001903. 10.1002/14651858.CD001903.pub5 32588435 PMC7387249

[31] Yuan X Wang J Yang S Gao M Cao L Li X . Effect of the ketogenic diet on glycemic control, insulin resistance, and lipid metabolism in patients with T2DM: a systematic review and meta-analysis. Nutr Diabetes. (2020) 10:38. 10.1038/s41387-020-00142-z 33257645 PMC7705738

[32] Yang YF Mattamel PB Joseph T Huang J Chen Q Akinwunmi BO . Efficacy of low-carbohydrate ketogenic diet as an adjuvant cancer therapy: a systematic review and meta-analysis of randomized controlled trials. Nutrients. (2021) 13:1388. 10.3390/nu13051388 33918992 PMC8142992

[33] Mayes PA Felts JM. Regulation of fat metabolism in the liver. Nature. (1967) 215:716–8. 10.1038/215716a0 6059540

[34] McGarry JD Foster DW. Hormonal control of ketogenesis. In: Hormones and Energy Metabolism. Boston, MA: Springer (1979), p. 79–96. 10.1007/978-1-4757-0734-2_4 371356

[35] McGarry JD Foster DW. Regulation of ketogenesis and clinical aspects of the ketotic state. Metabolism. (1972) 21:471–89. 10.1016/0026-0495(72)90059-5 4622681

[36] Robinson AM Williamson DH. Physiological roles of ketone bodies as substrates and signals in mammalian tissues. Physiol Rev. (1980) 60:143–87. 10.1152/physrev.1980.60.1.143 6986618

[37] Paoli A Rubini A Volek JS Grimaldi KA. Beyond weight loss: a review of the therapeutic uses of very-low-carbohydrate (ketogenic) diets. Eur J Clin Nutr. (2013) 67:789–6. 10.1038/ejcn.2013.116 23801097 PMC3826507

[38] Passmore R Johnson RE. The modification of post-exercise ketosis (the CourticeDouglas effect) by environmental temperature and water balance. Q J Exp Physiol. (1958) 43:352. 10.1113/expphysiol.1958.sp001348 13591502

[39] Prospective Studies Collaboration Whitlock G Lewington S Sherliker P Clarke R Emberson J . Body-mass index and cause-specific mortality in 900 000 adults: collaborative analyses of 57 prospective studies. Lancet. (2009) 373:1083–96. 10.1016/S0140-6736(09)60318-4 19299006 PMC2662372

[40] WCRF/AICR Food Nutrition Physical Activity and and the Prevention of Cancer: A Global Perspective. Washington, DC: AICR (2007).

[41] Prospective Studies Collaboration Lewington S Whitlock G Clarke R Sherliker P Emberson J . Blood cholesterol and vascular mortality by age, sex, and blood pressure: a meta-analysis of individual data from 61 prospective studies with 55,000 vascular deaths. Lancet. (2007) 370:1829–39. 10.1016/S0140-6736(07)61778-4 18061058

[42] Clarke R Frost C Collins R Appleby P Peto R. Dietary lipids and blood cholesterol: quantitative meta-analysis of metabolic ward studies. BMJ. (1997) 314:112–7. 10.1136/bmj.314.7074.112 9006469 PMC2125600

[43] EFSA. EFSA sets European dietary reference values for nutrient intakes | EFSA. Available at: https://www.efsa.europa.eu/en/press/news/nda100326 (accessed June 22, 2024).

[44] Scientific Opinion on Dietary Reference Values for carbohydrates and dietary fibre. EFSA J. (2016) 8:2–3. 10.2903/j.efsa.2010.1462

[45] Yang R Wen J Wei W Chen H Cao D Chen L . Improving the effects of ketogenic diet therapy in children with drug-resistant epilepsy. Seizure. (2022) 94:183–8. 10.1016/j.seizure.2021.10.021 34802897

[46] Ajala O English P Pinkney J. Systematic review and meta-analysis of different dietary approaches to the management of type 2 diabetes. Am J Clin Nutr. (2013) 97:505–16. 10.3945/ajcn.112.042457 23364002

