# Supplementary material for: Impact of low-carbohydrate diet on health status: an umbrella review
Source: Front Nutr. 2024 Sep 25;11:1321198. doi: 10.3389/fnut.2024.1321198 (PMC11462622; doi:10.3389/fnut.2024.1321198)
Supplement: Supplementary file 1 [file Table_1.DOCX]

**Excluded studies**

| **1.**Type 1 Diabetes Mellitus and Cognitive Impairments: A Systematic Review | **Li, W., Huang, E., & Gao, S. (2017). Type 1 Diabetes Mellitus and Cognitive Impairments: A Systematic Review. *Journal of Alzheimer's disease : JAD*, *57*(1), 29–36. https://doi.org/10.3233/JAD-161250** |
| --- | --- |
| **2.**Associations of Diet and Physical Activity with Risk for Gestational Diabetes Mellitus: A Systematic Review and Meta-Analysis | **Mijatovic-Vukas, J., Capling, L., Cheng, S., Stamatakis, E., Louie, J., Cheung, N. W., Markovic, T., Ross, G., Senior, A., Brand-Miller, J. C., & Flood, V. M. (2018). Associations of Diet and Physical Activity with Risk for Gestational Diabetes Mellitus: A Systematic Review and Meta-Analysis. Nutrients, 10(6), 698. https://doi.org/10.3390/nu10060698** |
| **3.**Dietary carbohydrate intake and mortality: a prospective cohort study and meta-analysis | **Seidelmann, S. B., Claggett, B., Cheng, S., Henglin, M., Shah, A., Steffen, L. M., Folsom, A. R., Rimm, E. B., Willett, W. C., & Solomon, S. D. (2018). Dietary carbohydrate intake and mortality: a prospective cohort study and meta-analysis. *The Lancet. Public health*, *3*(9), e419–e428. https://doi.org/10.1016/S2468-2667(18)30135-X** |
| **4.**The genetic architecture of the human cerebral cortex | **Grasby, K. L., Jahanshad, N., Painter, J. N., Colodro-Conde, L., Bralten, J., Hibar, D. P., Lind, P. A., Pizzagalli, F., Ching, C. R. K., McMahon, M. A. B., Shatokhina, N., Zsembik, L. C. P., Thomopoulos, S. I., Zhu, A. H., Strike, L. T., Agartz, I., Alhusaini, S., Almeida, M. A. A., Alnæs, D., Amlien, I. K., … Enhancing NeuroImaging Genetics through Meta-Analysis Consortium (ENIGMA)—Genetics working group (2020). The genetic architecture of the human cerebral cortex. *Science (New York, N.Y.)*, *367*(6484), eaay6690. https://doi.org/10.1126/science.aay6690** |
| **5.**A journey into a Mediterranean diet and type 2 diabetes: a systematic review with meta-analyses | **Esposito, K., Maiorino, M. I., Bellastella, G., Chiodini, P., Panagiotakos, D., & Giugliano, D. (2015). A journey into a Mediterranean diet and type 2 diabetes: a systematic review with meta-analyses. *BMJ open*, *5*(8), e008222. https://doi.org/10.1136/bmjopen-2015-008222** |
| **6.**Macronutrient Intake in Soccer Players-A Meta-Analysis | **Steffl, M., Kinkorova, I., Kokstejn, J., & Petr, M. (2019). Macronutrient Intake in Soccer Players-A Meta-Analysis. *Nutrients*, *11*(6), 1305. https://doi.org/10.3390/nu11061305** |
| **7.**A systematic review and meta-analysis of the nutrient content of preterm and term breast milk | **Gidrewicz, D. A., & Fenton, T. R. (2014). A systematic review and meta-analysis of the nutrient content of preterm and term breast milk. *BMC pediatrics*, *14*, 216. https://doi.org/10.1186/1471-2431-14-216** |
| **8.**Risks and Benefits of Chimeric Antigen Receptor T-Cell (CAR-T) Therapy in Cancer: A Systematic Review and Meta-Analysis | **Grigor, E. J. M., Fergusson, D., Kekre, N., Montroy, J., Atkins, H., Seftel, M. D., Daugaard, M., Presseau, J., Thavorn, K., Hutton, B., Holt, R. A., & Lalu, M. M. (2019). Risks and Benefits of Chimeric Antigen Receptor T-Cell (CAR-T) Therapy in Cancer: A Systematic Review and Meta-Analysis. *Transfusion medicine reviews*, *33*(2), 98–110. https://doi.org/10.1016/j.tmrv.2019.01.005** |
| **9.**Association of estimated glomerular filtration rate and albuminuria with all-cause and cardiovascular mortality in general population cohorts: a collaborative meta-analysis | **Chronic Kidney Disease Prognosis Consortium, Matsushita, K., van der Velde, M., Astor, B. C., Woodward, M., Levey, A. S., de Jong, P. E., Coresh, J., & Gansevoort, R. T. (2010). Association of estimated glomerular filtration rate and albuminuria with all-cause and cardiovascular mortality in general population cohorts: a collaborative meta-analysis. *Lancet (London, England)*, *375*(9731), 2073–2081. https://doi.org/10.1016/S0140-6736(10)60674-5** |
| **10.**Omega-3, omega-6, and total dietary polyunsaturated fat for prevention and treatment of type 2 diabetes mellitus: systematic review and meta-analysis of randomized controlled trials | **Brown, T. J., Brainard, J., Song, F., Wang, X., Abdelhamid, A., Hooper, L., & PUFAH Group (2019). Omega-3, omega-6, and total dietary polyunsaturated fat for prevention and treatment of type 2 diabetes mellitus: systematic review and meta-analysis of randomised controlled trials. *BMJ (Clinical research ed.)*, *366*, l4697. https://doi.org/10.1136/bmj.l4697** |
| **11.**Effects of Saturated Fat, Polyunsaturated Fat, Monounsaturated Fat, and Carbohydrate on Glucose-Insulin Homeostasis: A Systematic Review and Meta-analysis of Randomized Controlled Feeding Trials | **Imamura, F., Micha, R., Wu, J. H., de Oliveira Otto, M. C., Otite, F. O., Abioye, A. I., & Mozaffarian, D. (2016). Effects of Saturated Fat, Polyunsaturated Fat, Monounsaturated Fat, and Carbohydrate on Glucose-Insulin Homeostasis: A Systematic Review and Meta-analysis of Randomised Controlled Feeding Trials. *PLoS medicine*, *13*(7), e1002087. https://doi.org/10.1371/journal.pmed.1002087** |
| **12.**Meta-analysis of fecal metagenomes reveals global microbial signatures that are specific for colorectal cancer | **Wirbel, J., Pyl, P. T., Kartal, E., Zych, K., Kashani, A., Milanese, A., Fleck, J. S., Voigt, A. Y., Palleja, A., Ponnudurai, R., Sunagawa, S., Coelho, L. P., Schrotz-King, P., Vogtmann, E., Habermann, N., Niméus, E., Thomas, A. M., Manghi, P., Gandini, S., Serrano, D., … Zeller, G. (2019). Meta-analysis of fecal metagenomes reveals global microbial signatures that are specific for colorectal cancer. *Nature medicine*, *25*(4), 679–689. https://doi.org/10.1038/s41591-019-0406-6** |
| **13.**d-glutamate and Gut Microbiota in Alzheimer's Disease | **Chang, C. H., Lin, C. H., & Lane, H. Y. (2020). d-glutamate and Gut Microbiota in Alzheimer's Disease. *International journal of molecular sciences*, *21*(8), 2676. https://doi.org/10.3390/ijms21082676** |
| **14.**Individual NSAIDs and upper gastrointestinal complications: a systematic review and meta-analysis of observational studies (the SOS project) | **Castellsague, J., Riera-Guardia, N., Calingaert, B., Varas-Lorenzo, C., Fourrier-Reglat, A., Nicotra, F., Sturkenboom, M., Perez-Gutthann, S., & Safety of Non-Steroidal Anti-Inflammatory Drugs (SOS) Project (2012). Individual NSAIDs and upper gastrointestinal complications: a systematic review and meta-analysis of observational studies (the SOS project). *Drug safety*, *35*(12), 1127–1146. https://doi.org/10.2165/11633470-000000000-00000** |
| **15.**Reduction in saturated fat intake for cardiovascular disease | **Hooper, L., Martin, N., Abdelhamid, A., & Davey Smith, G. (2015). Reduction in saturated fat intake for cardiovascular disease. *The Cochrane database of systematic reviews*, (6), CD011737. https://doi.org/10.1002/14651858.CD011737** |
| **16.**Topical NSAIDs for chronic musculoskeletal pain in adults | **Yancey, J. R., & Gill, C. (2017). Topical NSAIDs for Chronic Musculoskeletal Pain in Adults. *American family physician*, *96*(9), 573–574.** |
| **17.**Conversion of Urine Protein-Creatinine Ratio or Urine Dipstick Protein to Urine Albumin-Creatinine Ratio for Use in Chronic Kidney Disease Screening and Prognosis: An Individual Participant-Based Meta-analysis | **Sumida, K., Nadkarni, G. N., Grams, M. E., Sang, Y., Ballew, S. H., Coresh, J., Matsushita, K., Surapaneni, A., Brunskill, N., Chadban, S. J., Chang, A. R., Cirillo, M., Daratha, K. B., Gansevoort, R. T., Garg, A. X., Iacoviello, L., Kayama, T., Konta, T., Kovesdy, C. P., Lash, J., … Chronic Kidney Disease Prognosis Consortium (2020). Conversion of Urine Protein-Creatinine Ratio or Urine Dipstick Protein to Urine Albumin-Creatinine Ratio for Use in Chronic Kidney Disease Screening and Prognosis : An Individual Participant-Based Meta-analysis. *Annals of internal medicine*, *173*(6), 426–435. https://doi.org/10.7326/M20-0529** |
| **18.**Associations of kidney disease measures with mortality and end-stage renal disease in individuals with and without diabetes: a meta-analysis | **Fox, C. S., Matsushita, K., Woodward, M., Bilo, H. J., Chalmers, J., Heerspink, H. J., Lee, B. J., Perkins, R. M., Rossing, P., Sairenchi, T., Tonelli, M., Vassalotti, J. A., Yamagishi, K., Coresh, J., de Jong, P. E., Wen, C. P., Nelson, R. G., & Chronic Kidney Disease Prognosis Consortium (2012). Associations of kidney disease measures with mortality and end-stage renal disease in individuals with and without diabetes: a meta-analysis. *Lancet (London, England)*, *380*(9854), 1662–1673. https://doi.org/10.1016/S0140-6736(12)61350-6** |
| **19.**Comparative safety of the sodium glucose co-transporter 2 (SGLT2) inhibitors: a systematic review and meta-analysis | **Donnan, J. R., Grandy, C. A., Chibrikov, E., Marra, C. A., Aubrey-Bassler, K., Johnston, K., Swab, M., Hache, J., Curnew, D., Nguyen, H., & Gamble, J. M. (2019). Comparative safety of the sodium glucose co-transporter 2 (SGLT2) inhibitors: a systematic review and meta-analysis. *BMJ open*, *9*(1), e022577. https://doi.org/10.1136/bmjopen-2018-022577** |
| **20.**Carbohydrate and fat intake associated with risk of metabolic diseases through epigenetics of CPT1A | **Lai, C. Q., Parnell, L. D., Smith, C. E., Guo, T., Sayols-Baixeras, S., Aslibekyan, S., Tiwari, H. K., Irvin, M. R., Bender, C., Fei, D., Hidalgo, B., Hopkins, P. N., Absher, D. M., Province, M. A., Elosua, R., Arnett, D. K., & Ordovas, J. M. (2020). Carbohydrate and fat intake associated with risk of metabolic diseases through epigenetics of CPT1A. *The American journal of clinical nutrition*, *112*(5), 1200–1211. https://doi.org/10.1093/ajcn/nqaa233** |
| **21.**Impact of different dietary approaches on glycemic control and cardiovascular risk factors in patients with type 2 diabetes: a protocol for a systematic review and network meta-analysis | **Schwingshackl, L., Chaimani, A., Hoffmann, G., Schwedhelm, C., & Boeing, H. (2017). Impact of different dietary approaches on glycemic control and cardiovascular risk factors in patients with type 2 diabetes: a protocol for a systematic review and network meta-analysis. *Systematic reviews*, *6*(1), 57. https://doi.org/10.1186/s13643-017-0455-1** |
| **22.**FTO genotype and weight loss in diet and lifestyle interventions: a systematic review and meta-analysis | **Xiang, L., Wu, H., Pan, A., Patel, B., Xiang, G., Qi, L., Kaplan, R. C., Hu, F., Wylie-Rosett, J., & Qi, Q. (2016). FTO genotype and weight loss in diet and lifestyle interventions: a systematic review and meta-analysis. *The American journal of clinical nutrition*, *103*(4), 1162–1170. https://doi.org/10.3945/ajcn.115.123448** |
| **23.**Topical NSAIDs for acute musculoskeletal pain in adults | **Derry, S., Moore, R. A., Gaskell, H., McIntyre, M., & Wiffen, P. J. (2015). Topical NSAIDs for acute musculoskeletal pain in adults. *The Cochrane database of systematic reviews*, *2015*(6), CD007402. https://doi.org/10.1002/14651858.CD007402.pub3** |
| **24.**Pharmacological and nutritional treatment for McArdle disease (Glycogen Storage Disease type V) | **Quinlivan, R., Martinuzzi, A., & Schoser, B. (2014). Pharmacological and nutritional treatment for McArdle disease (Glycogen Storage Disease type V). *The Cochrane database of systematic reviews*, *2014*(11), CD003458. https://doi.org/10.1002/14651858.CD003458.pub5** |
| **25.**The Energy Content and Composition of Meals Consumed after an Overnight Fast and Their Effects on Diet Induced Thermogenesis: A Systematic Review, Meta-Analyses and Meta-Regressions | **Quatela, A., Callister, R., Patterson, A., & MacDonald-Wicks, L. (2016). The Energy Content and Composition of Meals Consumed after an Overnight Fast and Their Effects on Diet Induced Thermogenesis: A Systematic Review, Meta-Analyses and Meta-Regressions. *Nutrients*, *8*(11), 670. https://doi.org/10.3390/nu8110670** |
| **26.**Interventions for treating oral leukoplakia to prevent oral cancer | **Greenslade R. (2017). Interventions for treating oral leukoplakia to prevent oral cancer. *Nursing standard (Royal College of Nursing (Great Britain) : 1987)*, *31*(39), 42–43. https://doi.org/10.7748/ns.2017.e10891** |
| **27.**Ketorolac for postoperative pain in children | **McNicol, E. D., Rowe, E., & Cooper, T. E. (2018). Ketorolac for postoperative pain in children. *The Cochrane database of systematic reviews*, *7*(7), CD012294. https://doi.org/10.1002/14651858.CD012294.pub2** |
| **28.**Benefits and Harms of Sodium-Glucose Co-Transporter 2 Inhibitors in Patients with Type 2 Diabetes: A Systematic Review and Meta-Analysis | **Storgaard, H., Gluud, L. L., Bennett, C., Grøndahl, M. F., Christensen, M. B., Knop, F. K., & Vilsbøll, T. (2016). Benefits and Harms of Sodium-Glucose Co-Transporter 2 Inhibitors in Patients with Type 2 Diabetes: A Systematic Review and Meta-Analysis. *PloS one*, *11*(11), e0166125. https://doi.org/10.1371/journal.pone.0166125** |
| **29.**Efficacy and safety of over-the-counter analgesics for primary dysmenorrhea: A network meta-analysis | **Nie, W., Xu, P., Hao, C., Chen, Y., Yin, Y., & Wang, L. (2020). Efficacy and safety of over-the-counter analgesics for primary dysmenorrhea: A network meta-analysis. *Medicine*, *99*(19), e19881. https://doi.org/10.1097/MD.0000000000019881** |
| **30.**Immune checkpoint inhibitor-induced Type 1 diabetes: a systematic review and meta-analysis | **Akturk, H. K., Kahramangil, D., Sarwal, A., Hoffecker, L., Murad, M. H., & Michels, A. W. (2019). Immune checkpoint inhibitor-induced Type 1 diabetes: a systematic review and meta-analysis. *Diabetic medicine : a journal of the British Diabetic Association*, *36*(9), 1075–1081. https://doi.org/10.1111/dme.14050** |
| **31.**High protein diet is of benefit for patients with type 2 diabetes: An updated meta-analysis | **Zhao, W. T., Luo, Y., Zhang, Y., Zhou, Y., & Zhao, T. T. (2018). High protein diet is of benefit for patients with type 2 diabetes: An updated meta-analysis. *Medicine*, *97*(46), e13149. https://doi.org/10.1097/MD.0000000000013149** |
| **32.**Reduction in saturated fat intake for cardiovascular disease | **Hooper, L., Martin, N., Jimoh, O. F., Kirk, C., Foster, E., & Abdelhamid, A. S. (2020). Reduction in saturated fat intake for cardiovascular disease. *The Cochrane database of systematic reviews*, *5*(5), CD011737. https://doi.org/10.1002/14651858.CD011737.pub2** |
| **33.**Nutritional supplementation for hip fracture aftercare in older people | **Avenell, A., & Handoll, H. H. (2005). Nutritional supplementation for hip fracture aftercare in older people. *The Cochrane database of systematic reviews*, (2), CD001880. https://doi.org/10.1002/14651858.CD001880.pub3** |
| **34.**Insulin and glucose-lowering agents for treating people with diabetes and chronic kidney disease | **Lo, C., Toyama, T., Wang, Y., Lin, J., Hirakawa, Y., Jun, M., Cass, A., Hawley, C. M., Pilmore, H., Badve, S. V., Perkovic, V., & Zoungas, S. (2018). Insulin and glucose-lowering agents for treating people with diabetes and chronic kidney disease. *The Cochrane database of systematic reviews*, *9*(9), CD011798. https://doi.org/10.1002/14651858.CD011798.pub2** |
| **35.**Effectiveness of high-intensity interval training on glycemic control and cardiorespiratory fitness in patients with type 2 diabetes: a systematic review and meta-analysis | **Liu, J. X., Zhu, L., Li, P. J., Li, N., & Xu, Y. B. (2019). Effectiveness of high-intensity interval training on glycemic control and cardiorespiratory fitness in patients with type 2 diabetes: a systematic review and meta-analysis. *Aging clinical and experimental research*, *31*(5), 575–593. https://doi.org/10.1007/s40520-018-1012-z** |
| **36.**FTO genotype and weight loss: systematic review and meta-analysis of 9563 individual participant data from eight randomized controlled trials | **Livingstone, K. M., Celis-Morales, C., Papandonatos, G. D., Erar, B., Florez, J. C., Jablonski, K. A., Razquin, C., Marti, A., Heianza, Y., Huang, T., Sacks, F. M., Svendstrup, M., Sui, X., Church, T. S., Jääskeläinen, T., Lindström, J., Tuomilehto, J., Uusitupa, M., Rankinen, T., Saris, W. H., … Mathers, J. C. (2016). FTO genotype and weight loss: systematic review and meta-analysis of 9563 individual participant data from eight randomised controlled trials. *BMJ (Clinical research ed.)*, *354*, i4707. https://doi.org/10.1136/bmj.i4707** |
| **37.**Clinical Efficacy of Stem Cell Therapy for Diabetes Mellitus: A Meta-Analysis | **El-Badawy, A., & El-Badri, N. (2016). Clinical Efficacy of Stem Cell Therapy for Diabetes Mellitus: A Meta-Analysis. *PloS one*, *11*(4), e0151938. https://doi.org/10.1371/journal.pone.0151938** |
| **38.**Exploring Opioid-Sparing Multimodal Analgesia Options in Trauma: A Nursing Perspective | **Sullivan, D., Lyons, M., Montgomery, R., & Quinlan-Colwell, A. (2016). Exploring Opioid-Sparing Multimodal Analgesia Options in Trauma: A Nursing Perspective. *Journal of trauma nursing : the official journal of the Society of Trauma Nurses*, *23*(6), 361–375. https://doi.org/10.1097/JTN.0000000000000250** |
| **39.**Phosphate binders for preventing and treating chronic kidney disease-mineral and bone disorder (CKD-MBD) | **Ruospo, M., Palmer, S. C., Natale, P., Craig, J. C., Vecchio, M., Elder, G. J., & Strippoli, G. F. (2018). Phosphate binders for preventing and treating chronic kidney disease-mineral and bone disorder (CKD-MBD). *The Cochrane database of systematic reviews*, *8*(8), CD006023. https://doi.org/10.1002/14651858.CD006023.pub3** |
| **40.**Assessing the risk of ketoacidosis due to sodium-glucose cotransporter (SGLT)-2 inhibitors in patients with type 1 diabetes: A meta-analysis and meta-regression | **Musso, G., Sircana, A., Saba, F., Cassader, M., & Gambino, R. (2020). Assessing the risk of ketoacidosis due to sodium-glucose cotransporter (SGLT)-2 inhibitors in patients with type 1 diabetes: A meta-analysis and meta-regression. *PLoS medicine*, *17*(12), e1003461. https://doi.org/10.1371/journal.pmed.1003461** |
| **41.**Resistance Training Prevents Muscle Loss Induced by Caloric Restriction in Obese Elderly Individuals: A Systematic Review and Meta-Analysis | **Sardeli, A. V., Komatsu, T. R., Mori, M. A., Gáspari, A. F., & Chacon-Mikahil, M. P. T. (2018). Resistance Training Prevents Muscle Loss Induced by Caloric Restriction in Obese Elderly Individuals: A Systematic Review and Meta-Analysis. *Nutrients*, *10*(4), 423. https://doi.org/10.3390/nu10040423** |
| **42.**Comparison of the efficacy and safety of non-steroidal anti-inflammatory drugs for patients with primary dysmenorrhea: A network meta-analysis | **Feng, X., & Wang, X. (2018). Comparison of the efficacy and safety of non-steroidal anti-inflammatory drugs for patients with primary dysmenorrhea: A network meta-analysis. *Molecular pain*, *14*, 1744806918770320. https://doi.org/10.1177/1744806918770320** |
| **43.**Continuous glucose monitoring systems for type 1 diabetes mellitus | **Langendam, M., Luijf, Y. M., Hooft, L., Devries, J. H., Mudde, A. H., & Scholten, R. J. (2012). Continuous glucose monitoring systems for type 1 diabetes mellitus. *The Cochrane database of systematic reviews*, *1*(1), CD008101. https://doi.org/10.1002/14651858.CD008101.pub2** |
| **44.**The Role of Dietary Fibre in Modulating Gut Microbiota Dysbiosis in Patients with Type 2 Diabetes: A Systematic Review and Meta-Analysis of Randomized Controlled Trials | **Ojo, O., Feng, Q. Q., Ojo, O. O., & Wang, X. H. (2020). The Role of Dietary Fibre in Modulating Gut Microbiota Dysbiosis in Patients with Type 2 Diabetes: A Systematic Review and Meta-Analysis of Randomised Controlled Trials. *Nutrients*, *12*(11), 3239. https://doi.org/10.3390/nu12113239** |
| **45.**Single-dose intravenous diclofenac for acute postoperative pain in adults | **McNicol, E. D., Ferguson, M. C., & Schumann, R. (2018). Single-dose intravenous diclofenac for acute postoperative pain in adults. *The Cochrane database of systematic reviews*, *8*(8), CD012498. https://doi.org/10.1002/14651858.CD012498.pub2** |
| **46.**Is Caloric Restriction Associated with Better Healthy Aging Outcomes? A Systematic Review and Meta-Analysis of Randomized Controlled Trials | **Caristia, S., Vito, M., Sarro, A., Leone, A., Pecere, A., Zibetti, A., Filigheddu, N., Zeppegno, P., Prodam, F., Faggiano, F., & Marzullo, P. (2020). Is Caloric Restriction Associated with Better Healthy Aging Outcomes? A Systematic Review and Meta-Analysis of Randomized Controlled Trials. *Nutrients*, *12*(8), 2290. https://doi.org/10.3390/nu12082290** |
| **47.**Paracetamol (acetaminophen) for acute treatment of episodic tension-type headache in adults | **Stephens, G., Derry, S., & Moore, R. A. (2016). Paracetamol (acetaminophen) for acute treatment of episodic tension-type headache in adults. *The Cochrane database of systematic reviews*, *2016*(6), CD011889. https://doi.org/10.1002/14651858.CD011889.pub2** |
| **48.**Systematic review of analgesics and dexamethasone for post-tonsillectomy pain in adults | **Tolska, H. K., Hamunen, K., Takala, A., & Kontinen, V. K. (2019). Systematic review of analgesics and dexamethasone for post-tonsillectomy pain in adults. *British journal of anaesthesia*, *123*(2), e397–e411. https://doi.org/10.1016/j.bja.2019.04.063** |
| **49.**Different types of dietary advice for women with gestational diabetes mellitus | **Han, S., Middleton, P., Shepherd, E., Van Ryswyk, E., & Crowther, C. A. (2017). Different types of dietary advice for women with gestational diabetes mellitus. *The Cochrane database of systematic reviews*, *2*(2), CD009275. https://doi.org/10.1002/14651858.CD009275.pub3** |
| **50.**Topical antifungals for seborrhoeic dermatitis | **Okokon, E. O., Verbeek, J. H., Ruotsalainen, J. H., Ojo, O. A., & Bakhoya, V. N. (2015). Topical antifungals for seborrhoeic dermatitis. *The Cochrane database of systematic reviews*, (5), CD008138. https://doi.org/10.1002/14651858.CD008138.pub3** |
| **51.**Genetic predisposition to hypertension is associated with preeclampsia in European and Central Asian women | **Steinthorsdottir, V., McGinnis, R., Williams, N. O., Stefansdottir, L., Thorleifsson, G., Shooter, S., Fadista, J., Sigurdsson, J. K., Auro, K. M., Berezina, G., Borges, M. C., Bumpstead, S., Bybjerg-Grauholm, J., Colgiu, I., Dolby, V. A., Dudbridge, F., Engel, S. M., Franklin, C. S., Frigge, M. L., Frisbaek, Y., … Morgan, L. (2020). Genetic predisposition to hypertension is associated with preeclampsia in European and Central Asian women. *Nature communications*, *11*(1), 5976. https://doi.org/10.1038/s41467-020-19733-6** |
| **52.**Intensive glucose control versus conventional glucose control for type 1 diabetes mellitus | **Fullerton, B., Jeitler, K., Seitz, M., Horvath, K., Berghold, A., & Siebenhofer, A. (2014). Intensive glucose control versus conventional glucose control for type 1 diabetes mellitus. *The Cochrane database of systematic reviews*, *2014*(2), CD009122. https://doi.org/10.1002/14651858.CD009122.pub2** |
| **53.**New insulin delivery devices and glycemic outcomes in young patients with type 1 diabetes: a protocol for a systematic review and meta-analysis | **Dos Santos, T. J., Donado Campos, J. M., Fraga Medin, C. A., Argente, J., & Rodríguez-Artalejo, F. (2019). New insulin delivery devices and glycemic outcomes in young patients with type 1 diabetes: a protocol for a systematic review and meta-analysis. *Systematic reviews*, *8*(1), 259. https://doi.org/10.1186/s13643-019-1171-9** |
| **54.**Estimated glomerular filtration rate and albuminuria for prediction of cardiovascular outcomes: a collaborative meta-analysis of individual participant data | **Matsushita, K., Coresh, J., Sang, Y., Chalmers, J., Fox, C., Guallar, E., Jafar, T., Jassal, S. K., Landman, G. W., Muntner, P., Roderick, P., Sairenchi, T., Schöttker, B., Shankar, A., Shlipak, M., Tonelli, M., Townend, J., van Zuilen, A., Yamagishi, K., Yamashita, K., … CKD Prognosis Consortium (2015). Estimated glomerular filtration rate and albuminuria for prediction of cardiovascular outcomes: a collaborative meta-analysis of individual participant data. *The lancet. Diabetes & endocrinology*, *3*(7), 514–525. https://doi.org/10.1016/S2213-8587(15)00040-6** |
| **55.**Cystatin C versus creatinine in determining risk based on kidney function | **Shlipak, M. G., Matsushita, K., Ärnlöv, J., Inker, L. A., Katz, R., Polkinghorne, K. R., Rothenbacher, D., Sarnak, M. J., Astor, B. C., Coresh, J., Levey, A. S., Gansevoort, R. T., & CKD Prognosis Consortium (2013). Cystatin C versus creatinine in determining risk based on kidney function. *The New England journal of medicine*, *369*(10), 932–943. https://doi.org/10.1056/NEJMoa1214234** |
| **56.**Single dose oral ketoprofen or dexketoprofen for acute postoperative pain in adults | **Barden, J., Derry, S., McQuay, H. J., & Moore, R. A. (2009). Single dose oral ketoprofen and dexketoprofen for acute postoperative pain in adults. *The Cochrane database of systematic reviews*, (4), CD007355. https://doi.org/10.1002/14651858.CD007355.pub2** |
| **57.**Efficacy and safety of dual SGLT 1/2 inhibitor sotagliflozin in type 1 diabetes: meta-analysis of randomised controlled trials | **Musso, G., Gambino, R., Cassader, M., & Paschetta, E. (2019). Efficacy and safety of dual SGLT 1/2 inhibitor sotagliflozin in type 1 diabetes: meta-analysis of randomised controlled trials. *BMJ (Clinical research ed.)*, *365*, l1328. https://doi.org/10.1136/bmj.l1328** |
| **58.**Large meta-analysis of genome-wide association studies identifies five loci for lean body mass | **Zillikens, M. C., Demissie, S., Hsu, Y. H., Yerges-Armstrong, L. M., Chou, W. C., Stolk, L., Livshits, G., Broer, L., Johnson, T., Koller, D. L., Kutalik, Z., Luan, J., Malkin, I., Ried, J. S., Smith, A. V., Thorleifsson, G., Vandenput, L., Hua Zhao, J., Zhang, W., Aghdassi, A., … Kiel, D. P. (2017). Large meta-analysis of genome-wide association studies identifies five loci for lean body mass. *Nature communications*, *8*(1), 80. https://doi.org/10.1038/s41467-017-00031-7** |
| **59.**Dapagliflozin in patients with type 2 diabetes mellitus: A pooled analysis of safety data from phase IIb/III clinical trials | **Jabbour, S., Seufert, J., Scheen, A., Bailey, C. J., Karup, C., & Langkilde, A. M. (2018). Dapagliflozin in patients with type 2 diabetes mellitus: A pooled analysis of safety data from phase IIb/III clinical trials. *Diabetes, obesity & metabolism*, *20*(3), 620–628. https://doi.org/10.1111/dom.13124** |
| **60.**The Effect of Ketoanalogues on Chronic Kidney Disease Deterioration: A Meta-Analysis | **Li, A., Lee, H. Y., & Lin, Y. C. (2019). The Effect of Ketoanalogues on Chronic Kidney Disease Deterioration: A Meta-Analysis. *Nutrients*, *11*(5), 957. https://doi.org/10.3390/nu11050957** |
| **61.**Single dose dipyrone (metamizole) for acute postoperative pain in adults | **Hearn, L., Derry, S., & Moore, R. A. (2016). Single dose dipyrone (metamizole) for acute postoperative pain in adults. *The Cochrane database of systematic reviews*, *4*(4), CD011421. https://doi.org/10.1002/14651858.CD011421.pub2** |
| **62.**Decline in estimated glomerular filtration rate and subsequent risk of end-stage renal disease and mortality | **Coresh, J., Turin, T. C., Matsushita, K., Sang, Y., Ballew, S. H., Appel, L. J., Arima, H., Chadban, S. J., Cirillo, M., Djurdjev, O., Green, J. A., Heine, G. H., Inker, L. A., Irie, F., Ishani, A., Ix, J. H., Kovesdy, C. P., Marks, A., Ohkubo, T., Shalev, V., … Levey, A. S. (2014). Decline in estimated glomerular filtration rate and subsequent risk of end-stage renal disease and mortality. *JAMA*, *311*(24), 2518–2531. https://doi.org/10.1001/jama.2014.6634** |
| **63.**Prophylactic non-steroidal anti-inflammatory drugs for the prevention of macular oedema after cataract surgery | **Lim, B. X., Lim, C. H., Lim, D. K., Evans, J. R., Bunce, C., & Wormald, R. (2016). Prophylactic non-steroidal anti-inflammatory drugs for the prevention of macular oedema after cataract surgery. *The Cochrane database of systematic reviews*, *11*(11), CD006683. https://doi.org/10.1002/14651858.CD006683.pub3** |
| **64.**Early enteral nutrition within 24 hours of lower gastrointestinal surgery versus later commencement for length of hospital stay and postoperative complications | **Stannard D. (2020). Early Enteral Nutrition Within 24 Hours of Lower Gastrointestinal Surgery Versus Later Commencement for Length of Hospital Stay and Postoperative Complications. *Journal of perianesthesia nursing : official journal of the American Society of PeriAnesthesia Nurses*, *35*(5), 541–542. https://doi.org/10.1016/j.jopan.2020.07.003** |
| **65.**High-carbohydrate, high-protein, low-fat versus low-carbohydrate, high-protein, high-fat enteral feeds for burns | **Masters, B., Aarabi, S., Sidhwa, F., & Wood, F. (2012). High-carbohydrate, high-protein, low-fat versus low-carbohydrate, high-protein, high-fat enteral feeds for burns. *The Cochrane database of systematic reviews*, *1*, CD006122. https://doi.org/10.1002/14651858.CD006122.pub3** |
| **66.**A meta-analysis on associations of FTO, MTHFR and TCF7L2 polymorphisms with polycystic ovary syndrome | **Wang, X., Wang, K., Yan, J., & Wu, M. (2020). A meta-analysis on associations of FTO, MTHFR and TCF7L2 polymorphisms with polycystic ovary syndrome. *Genomics*, *112*(2), 1516–1521. https://doi.org/10.1016/j.ygeno.2019.08.023** |
| **67.**A systematic review and meta-analysis of cold in situ perfusion and preservation for pancreas transplantation | **Hameed, A. M., Wong, G., Laurence, J. M., Lam, V. W. T., Pleass, H. C., & Hawthorne, W. J. (2017). A systematic review and meta-analysis of cold in situ perfusion and preservation for pancreas transplantation. *HPB : the official journal of the International Hepato Pancreato Biliary Association*, *19*(11), 933–943. https://doi.org/10.1016/j.hpb.2017.07.012** |
| **68.**Exercise, adipokines and pediatric obesity: a meta-analysis of randomized controlled trials | **García-Hermoso, A., Ceballos-Ceballos, R. J., Poblete-Aro, C. E., Hackney, A. C., Mota, J., & Ramírez-Vélez, R. (2017). Exercise, adipokines and pediatric obesity: a meta-analysis of randomized controlled trials. *International journal of obesity (2005)*, *41*(4), 475–482. https://doi.org/10.1038/ijo.2016.230** |
| **69.**Macronutrient Intake and Risk of Crohn's Disease: Systematic Review and Dose-Response Meta-Analysis of Epidemiological Studies | **Zeng, L., Hu, S., Chen, P., Wei, W., & Tan, Y. (2017). Macronutrient Intake and Risk of Crohn's Disease: Systematic Review and Dose-Response Meta-Analysis of Epidemiological Studies. *Nutrients*, *9*(5), 500. https://doi.org/10.3390/nu9050500** |
| **70.**Effects of Ramadan Observance on Dietary Intake and Body Composition of Adolescent Athletes: Systematic Review and Meta-Analysis | **Trabelsi, K., Ammar, A., Boukhris, O., Glenn, J. M., Bott, N., Stannard, S. R., Engel, F. A., Sperlich, B., Garbarino, S., Bragazzi, N. L., Shephard, R. J., & Chtourou, H. (2020). Effects of Ramadan Observance on Dietary Intake and Body Composition of Adolescent Athletes: Systematic Review and Meta-Analysis. *Nutrients*, *12*(6), 1574. https://doi.org/10.3390/nu12061574** |
| **71.**Meta-analysis of the relationship between milk trans-10 C18:1, milk fatty acids <16 C, and milk fat production | **Matamoros, C., Klopp, R. N., Moraes, L. E., & Harvatine, K. J. (2020). Meta-analysis of the relationship between milk trans-10 C18:1, milk fatty acids <16 C, and milk fat production. *Journal of dairy science*, *103*(11), 10195–10206. https://doi.org/10.3168/jds.2019-18129** |
| **72.**Meta-analysis of factors that affect the utilization efficiency of phosphorus in lactating dairy cows | **Klop, G., Ellis, J. L., Bannink, A., Kebreab, E., France, J., & Dijkstra, J. (2013). Meta-analysis of factors that affect the utilization efficiency of phosphorus in lactating dairy cows. *Journal of dairy science*, *96*(6), 3936–3949. https://doi.org/10.3168/jds.2012-6336** |
| **73.**Ketamine-propofol (Ketofol) for procedural sedation and analgesia in children: a systematic review and meta-analysis | **Foo, T. Y., Mohd Noor, N., Yazid, M. B., Fauzi, M. H., Abdull Wahab, S. F., & Ahmad, M. Z. (2020). Ketamine-propofol (Ketofol) for procedural sedation and analgesia in children: a systematic review and meta-analysis. *BMC emergency medicine*, *20*(1), 81. https://doi.org/10.1186/s12873-020-00373-4** |
| **74.**Gene-diet interaction of FTO-rs9939609 gene variant and hypocaloric diet on glycemic control in overweight and obese adults: a systematic review and meta-analysis of clinical trials | **Parastouei, K., Rostami, H., Ramezani, A. A., Tavakoli, H., & Alipour, M. (2020). Gene-diet interaction of FTO-rs9939609 gene variant and hypocaloric diet on glycemic control in overweight and obese adults: a systematic review and meta-analysis of clinical trials. *Chinese medical journal*, *133*(3), 310–317. https://doi.org/10.1097/CM9.0000000000000617** |
| **75.**Medium Chain Triglycerides induce mild ketosis and may improve cognition in Alzheimer's disease. A systematic review and meta-analysis of human studies | **Avgerinos, K. I., Egan, J. M., Mattson, M. P., & Kapogiannis, D. (2020). Medium Chain Triglycerides induce mild ketosis and may improve cognition in Alzheimer's disease. A systematic review and meta-analysis of human studies. *Ageing research reviews*, *58*, 101001. https://doi.org/10.1016/j.arr.2019.101001** |
| **76.**The effects of various diets on glycemic outcomes during pregnancy: A systematic review and network meta-analysis | **Ha, V., Bonner, A. J., Jadoo, J. K., Beyene, J., Anand, S. S., & de Souza, R. J. (2017). The effects of various diets on glycemic outcomes during pregnancy: A systematic review and network meta-analysis. PloS one, 12(8), e0182095. https://doi.org/10.1371/journal.pone.0182095** |
| **77.**Genetic Predisposition to an Impaired Metabolism of the Branched-Chain Amino Acids and Risk of Type 2 Diabetes: A Mendelian Randomisation Analysis | **Lotta, L. A., Scott, R. A., Sharp, S. J., Burgess, S., Luan, J., Tillin, T., Schmidt, A. F., Imamura, F., Stewart, I. D., Perry, J. R., Marney, L., Koulman, A., Karoly, E. D., Forouhi, N. G., Sjögren, R. J., Näslund, E., Zierath, J. R., Krook, A., Savage, D. B., Griffin, J. L., … Langenberg, C. (2016). Genetic Predisposition to an Impaired Metabolism of the Branched-Chain Amino Acids and Risk of Type 2 Diabetes: A Mendelian Randomisation Analysis. *PLoS medicine*, *13*(11), e1002179. https://doi.org/10.1371/journal.pmed.1002179** |
| **78.**[NUTRIENTS AND RADIOTHERAPY; REVIEW OF THE LITERATURE] | **Luna, J., Amaya, E., de Torres, M. V., Peña, M. C., & Prieto, I. (2015). NUTRIENTES Y RADIOTERAPIA; REVISIÓN DE LA LITERATURA [NUTRIENTS AND RADIOTHERAPY; REVIEW OF THE LITERATURE]. *Nutricion hospitalaria*, *32*(6), 2446–2459. https://doi.org/10.3305/nh.2015.32.6.9596** |
| **79.**A systematic review and meta-analysis of cold in situ perfusion and preservation of the hepatic allograft: Working toward a unified approach | **Hameed, A. M., Laurence, J. M., Lam, V. W. T., Pleass, H. C., & Hawthorne, W. J. (2017). A systematic review and meta-analysis of cold in situ perfusion and preservation of the hepatic allograft: Working toward a unified approach. Liver transplantation : official publication of the American Association for the Study of Liver Diseases and the International Liver Transplantation Society, 23(12), 1615–1627. https://doi.org/10.1002/lt.24829** |
| **80.**Meta-analysis of the effects of supplemental rumen-protected choline during the transition period on performance and health of parous dairy cows | **Arshad, U., Zenobi, M. G., Staples, C. R., & Santos, J. E. P. (2020). Meta-analysis of the effects of supplemental rumen-protected choline during the transition period on performance and health of parous dairy cows. Journal of dairy science, 103(1), 282–300. https://doi.org/10.3168/jds.2019-16842** |
| **81.**Meta-analysis of drainage versus no drainage after laparoscopic cholecystectomy | **Picchio, M., Lucarelli, P., Di Filippo, A., De Angelis, F., Stipa, F., & Spaziani, E. (2014). Meta-analysis of drainage versus no drainage after laparoscopic cholecystectomy. JSLS : Journal of the Society of Laparoendoscopic Surgeons, 18(4), e2014.00242. https://doi.org/10.4293/JSLS.2014.00242** |
| **82.**Association between circulating neuregulin4 levels and diabetes mellitus: A meta-analysis of observational studies | **Wang, Y., Huang, S., & Yu, P. (2019). Association between circulating neuregulin4 levels and diabetes mellitus: A meta-analysis of observational studies. PloS one, 14(12), e0225705. https://doi.org/10.1371/journal.pone.0225705** |
| **83.**Interaction between the FTO gene, body mass index and depression: meta-analysis of 13701 individuals | **Rivera, M., Locke, A. E., Corre, T., Czamara, D., Wolf, C., Ching-Lopez, A., Milaneschi, Y., Kloiber, S., Cohen-Woods, S., Rucker, J., Aitchison, K. J., Bergmann, S., Boomsma, D. I., Craddock, N., Gill, M., Holsboer, F., Hottenga, J. J., Korszun, A., Kutalik, Z., Lucae, S., … McGuffin, P. (2017). Interaction between the FTO gene, body mass index and depression: meta-analysis of 13701 individuals. The British journal of psychiatry : the journal of mental science, 211(2), 70–76. https://doi.org/10.1192/bjp.bp.116.183475** |
| **84.**Long-term effects of low-fat diets either low or high in protein on cardiovascular and metabolic risk factors: a systematic review and meta-analysis | **Schwingshackl, L., & Hoffmann, G. (2013). Long-term effects of low-fat diets either low or high in protein on cardiovascular and metabolic risk factors: a systematic review and meta-analysis. Nutrition journal, 12, 48. https://doi.org/10.1186/1475-2891-12-48** |
| **85.**Association between fat mass and obesity associated (FTO) gene rs9939609 A/T polymorphism and polycystic ovary syndrome: a systematic review and meta-analysis | **Liu, A. L., Xie, H. J., Xie, H. Y., Liu, J., Yin, J., Hu, J. S., & Peng, C. Y. (2017). Association between fat mass and obesity associated (FTO) gene rs9939609 A/T polymorphism and polycystic ovary syndrome: a systematic review and meta-analysis. BMC medical genetics, 18(1), 89. https://doi.org/10.1186/s12881-017-0452-1** |
| **86.**Ketoconazole associated hepatotoxicity: a systematic review and meta- analysis | **Yan, J. Y., Nie, X. L., Tao, Q. M., Zhan, S. Y., & Zhang, Y. D. (2013). Ketoconazole associated hepatotoxicity: a systematic review and meta- analysis. Biomedical and environmental sciences : BES, 26(7), 605–610. https://doi.org/10.3967/0895-3988.2013.07.013** |
| **87.**Pain control in first trimester surgical abortion | **Renner, R. M., Jensen, J. T., Nichols, M. D., & Edelman, A. (2009). Pain control in first trimester surgical abortion. The Cochrane database of systematic reviews, 2009(2), CD006712. https://doi.org/10.1002/14651858.CD006712.pub2** |
| **88.**Azoles for allergic bronchopulmonary aspergillosis associated with asthma | **Wark, P. A., Gibson, P. G., & Wilson, A. J. (2004). Azoles for allergic bronchopulmonary aspergillosis associated with asthma. The Cochrane database of systematic reviews, 2004(3), CD001108. https://doi.org/10.1002/14651858.CD001108.pub2** |
| **89.**Statistical modeling of ruminal pH parameters from dairy cows based on a meta-analysis | **Mensching, A., Hummel, J., & Sharifi, A. R. (2020). Statistical modeling of ruminal pH parameters from dairy cows based on a meta-analysis. Journal of dairy science, 103(1), 750–767. https://doi.org/10.3168/jds.2019-16802** |
| **90.**An Evaluation of the Nutritional Value and Physical Properties of Blenderised Enteral Nutrition Formula: A Systematic Review and Meta-Analysis | **Ojo, O., Adegboye, A. R. A., Ojo, O. O., Wang, X., & Brooke, J. (2020). An Evaluation of the Nutritional Value and Physical Properties of Blenderised Enteral Nutrition Formula: A Systematic Review and Meta-Analysis. Nutrients, 12(6), 1840. https://doi.org/10.3390/nu12061840** |
| **91.**Effect direction meta-analysis of GWAS identifies extreme, prevalent and shared pleiotropy in a large mammal | **Xiang, R., van den Berg, I., MacLeod, I. M., Daetwyler, H. D., & Goddard, M. E. (2020). Effect direction meta-analysis of GWAS identifies extreme, prevalent and shared pleiotropy in a large mammal. Communications biology, 3(1), 88. https://doi.org/10.1038/s42003-020-0823-6** |
| **92.**FTO gene variant and risk of overweight and obesity among children and adolescents: a systematic review and meta-analysis | **Liu, C., Mou, S., & Cai, Y. (2013). FTO gene variant and risk of overweight and obesity among children and adolescents: a systematic review and meta-analysis. PloS one, 8(11), e82133. https://doi.org/10.1371/journal.pone.0082133** |
| **93.**Efficacy and safety of ketoconazole combined with calmodulin inhibitor in solid organ transplantation: A systematic review and meta-analysis | **Xue, T., Yang, T., Chen, C., Wu, S., Li, M., Ma, L., Zhou, Y., & Cui, Y. (2020). Efficacy and safety of ketoconazole combined with calmodulin inhibitor in solid organ transplantation: A systematic review and meta-analysis. Journal of clinical pharmacy and therapeutics, 45(1), 29–34. https://doi.org/10.1111/jcpt.13043** |
| **94.**Associations of estimated glomerular filtration rate and albuminuria with mortality and renal failure by sex: a meta-analysis | **Nitsch, D., Grams, M., Sang, Y., Black, C., Cirillo, M., Djurdjev, O., Iseki, K., Jassal, S. K., Kimm, H., Kronenberg, F., Oien, C. M., Levey, A. S., Levin, A., Woodward, M., Hemmelgarn, B. R., & Chronic Kidney Disease Prognosis Consortium (2013). Associations of estimated glomerular filtration rate and albuminuria with mortality and renal failure by sex: a meta-analysis. BMJ (Clinical research ed.), 346, f324. https://doi.org/10.1136/bmj.f324** |
| **95.**Interventions for treating oral leukoplakia | **Lodi, G., Sardella, A., Bez, C., Demarosi, F., & Carrassi, A. (2006). Interventions for treating oral leukoplakia. The Cochrane database of systematic reviews, (4), CD001829. https://doi.org/10.1002/14651858.CD001829.pub3** |
| **96.**Treatment of delusions in Alzheimer's disease--response to pharmacotherapy | **Fischer, C., Bozanovic, R., Atkins, J. H., & Rourke, S. B. (2006). Treatment of delusions in Alzheimer's disease--response to pharmacotherapy. Dementia and geriatric cognitive disorders, 22(3), 260–266. https://doi.org/10.1159/000094975** |
| **97.**Short communication: Meta-analysis of dairy cows fed conventional sorghum or corn silages compared with brown midrib sorghum silage | **Sánchez-Duarte, J. I., Kalscheur, K. F., García, A. D., & Contreras-Govea, F. E. (2019). Short communication: Meta-analysis of dairy cows fed conventional sorghum or corn silages compared with brown midrib sorghum silage. *Journal of dairy science*, *102*(1), 419–425. https://doi.org/10.3168/jds.2018-14552** |
| **98.**Efficacy of azole therapy for tegumentary leishmaniasis: A systematic review and meta-analysis | **Galvão, E. L., Rabello, A., & Cota, G. F. (2017). Efficacy of azole therapy for tegumentary leishmaniasis: A systematic review and meta-analysis. PloS one, 12(10), e0186117. https://doi.org/10.1371/journal.pone.0186117** |
| **99.**Antiepileptic drugs for the treatment of infants with severe myoclonic epilepsy | **Brigo, F., & Igwe, S. C. (2015). Antiepileptic drugs for the treatment of infants with severe myoclonic epilepsy. The Cochrane database of systematic reviews, (10), CD010483. https://doi.org/10.1002/14651858.CD010483.pub3** |
| **100.**Efficacy and safety of sotagliflozin adjuvant therapy for type 1 diabetes mellitus: A systematic review and meta-analysis | **Chen, M. B., Xu, R. J., Zheng, Q. H., Zheng, X. W., & Wang, H. (2020). Efficacy and safety of sotagliflozin adjuvant therapy for type 1 diabetes mellitus: A systematic review and meta-analysis. Medicine, 99(33), e20875. https://doi.org/10.1097/MD.0000000000020875** |
| **101.**Clomiphene and other antioestrogens for ovulation induction in polycystic ovarian syndrome | **Brown, J., & Farquhar, C. (2016). Clomiphene and other antioestrogens for ovulation induction in polycystic ovarian syndrome. The Cochrane database of systematic reviews, 12(12), CD002249. https://doi.org/10.1002/14651858.CD002249.pub5** |
| **102.**Low protein diets are mainstay for management of chronic kidney disease | **De Santo, N. G., Perna, A., & Cirillo, M. (2011). Low protein diets are mainstay for management of chronic kidney disease. *Frontiers in bioscience (Scholar edition)*, *3*(4), 1432–1442. https://doi.org/10.2741/234** |
| **103.**Economic compensation interventions to increase uptake of voluntary medical male circumcision for HIV prevention: A systematic review and meta-analysis | **Kennedy, C. E., Yeh, P. T., Atkins, K., Fonner, V. A., Sweat, M. D., O'Reilly, K. R., Rutherford, G. W., Baggaley, R., & Samuelson, J. (2020). Economic compensation interventions to increase uptake of voluntary medical male circumcision for HIV prevention: A systematic review and meta-analysis. *PloS one*, *15*(1), e0227623. https://doi.org/10.1371/journal.pone.0227623** |
| **104.**[Systematic review to assess the effectiveness and safety of parecoxib] | **Villasís-Keever, M. A., Rendón-Macías, M. E., & Escamilla-Núñez, A. (2009). Revisi6n sistemática para determinar la efectividad y seguridad de parecoxib [Systematic review to assess the effectiveness and safety of parecoxib]. *Acta ortopedica mexicana*, *23*(6), 342–350.** |
| **105.**Diet fermentability influences lactational performance responses to corn distillers grains: a meta-analysis | **Hollmann, M., Allen, M. S., & Beede, D. K. (2011). Diet fermentability influences lactational performance responses to corn distillers grains: a meta-analysis. *Journal of dairy science*, *94*(4), 2007–2021. https://doi.org/10.3168/jds.2010-3711** |
| **106.**Oxcarbazepine add-on for drug-resistant focal epilepsy | **Bresnahan, R., Atim-Oluk, M., & Marson, A. G. (2020). Oxcarbazepine add-on for drug-resistant focal epilepsy. *The Cochrane database of systematic reviews*, *3*(3), CD012433. https://doi.org/10.1002/14651858.CD012433.pub2** |
| **107.**Adherence of Malaysian Adults' Energy and Macronutrient Intakes to National Recommendations: A Review and Meta-Analysis | **Shahar, S., Jan Bin Jan Mohamed, H., de Los Reyes, F., & Amarra, M. S. (2018). Adherence of Malaysian Adults' Energy and Macronutrient Intakes to National Recommendations: A Review and Meta-Analysis. *Nutrients*, *10*(11), 1584. https://doi.org/10.3390/nu10111584** |
| **108.**Associations of kidney disease measures with mortality and end-stage renal disease in individuals with and without hypertension: a meta-analysis | **Mahmoodi, B. K., Matsushita, K., Woodward, M., Blankestijn, P. J., Cirillo, M., Ohkubo, T., Rossing, P., Sarnak, M. J., Stengel, B., Yamagishi, K., Yamashita, K., Zhang, L., Coresh, J., de Jong, P. E., Astor, B. C., & Chronic Kidney Disease Prognosis Consortium (2012). Associations of kidney disease measures with mortality and end-stage renal disease in individuals with and without hypertension: a meta-analysis. *Lancet (London, England)*, *380*(9854), 1649–1661. https://doi.org/10.1016/S0140-6736(12)61272-0** |
| **109.**The FTO gene rs9939609 polymorphism predicts risk of cardiovascular disease: a systematic review and meta-analysis | **Liu, C., Mou, S., & Pan, C. (2013). The FTO gene rs9939609 polymorphism predicts risk of cardiovascular disease: a systematic review and meta-analysis. *PloS one*, *8*(8), e71901. https://doi.org/10.1371/journal.pone.0071901** |
| **110.**Genome-wide meta-analysis of macronutrient intake of 91,114 European ancestry participants from the cohorts for heart and aging research in genomic epidemiology consortium | **Merino, J., Dashti, H. S., Li, S. X., Sarnowski, C., Justice, A. E., Graff, M., Papoutsakis, C., Smith, C. E., Dedoussis, G. V., Lemaitre, R. N., Wojczynski, M. K., Männistö, S., Ngwa, J. S., Kho, M., Ahluwalia, T. S., Pervjakova, N., Houston, D. K., Bouchard, C., Huang, T., Orho-Melander, M., … Tanaka, T. (2019). Genome-wide meta-analysis of macronutrient intake of 91,114 European ancestry participants from the cohorts for heart and aging research in genomic epidemiology consortium. *Molecular psychiatry*, *24*(12), 1920–1932. https://doi.org/10.1038/s41380-018-0079-4** |
| **111.**Antifungal agents for preventing fungal infections in non-neutropenic critically ill patients | **Cortegiani, A., Russotto, V., Maggiore, A., Attanasio, M., Naro, A. R., Raineri, S. M., & Giarratano, A. (2016). Antifungal agents for preventing fungal infections in non-neutropenic critically ill patients. *The Cochrane database of systematic reviews*, *2016*(1), CD004920. https://doi.org/10.1002/14651858.CD004920.pub3** |
| **112.**FTO gene polymorphisms and obesity risk: a meta-analysis | **Peng, S., Zhu, Y., Xu, F., Ren, X., Li, X., & Lai, M. (2011). FTO gene polymorphisms and obesity risk: a meta-analysis. *BMC medicine*, *9*, 71. https://doi.org/10.1186/1741-7015-9-71** |
| **113.**Topical NSAIDs for acute pain in adults | **Massey, T., Derry, S., Moore, R. A., & McQuay, H. J. (2010). Topical NSAIDs for acute pain in adults. *The Cochrane database of systematic reviews*, (6), CD007402. https://doi.org/10.1002/14651858.CD007402.pub2** |
| **114.**The influence of topical non-steroidal anti-inflammatory drugs on the intraocular pressure lowering effect of topical prostaglandin analogues-A systemic review and meta-analysis | **Lo, K. J., Ko, Y. C., Hwang, D. K., & Liu, C. J. (2020). The influence of topical non-steroidal anti-inflammatory drugs on the intraocular pressure lowering effect of topical prostaglandin analogues-A systemic review and meta-analysis. *PloS one*, *15*(9), e0239233. https://doi.org/10.1371/journal.pone.0239233** |
| **115.**Anti-Tumor Effects of Ketogenic Diets in Mice: A Meta-Analysis | **Klement, R. J., Champ, C. E., Otto, C., & Kämmerer, U. (2016). Anti-Tumor Effects of Ketogenic Diets in Mice: A Meta-Analysis. *PloS one*, *11*(5), e0155050. https://doi.org/10.1371/journal.pone.0155050** |
| **116.**Metabolic effects of testosterone replacement therapy on hypogonadal men with type 2 diabetes mellitus: a systematic review and meta-analysis of randomized controlled trials | **Cai, X., Tian, Y., Wu, T., Cao, C. X., Li, H., & Wang, K. J. (2014). Metabolic effects of testosterone replacement therapy on hypogonadal men with type 2 diabetes mellitus: a systematic review and meta-analysis of randomized controlled trials. *Asian journal of andrology*, *16*(1), 146–152. https://doi.org/10.4103/1008-682X.122346** |
| **117.**Meal analysis for understanding eating behavior: meal- and participant-specific predictors for the variance in energy and macronutrient intake | **Schwedhelm, C., Iqbal, K., Schwingshackl, L., Agogo, G. O., Boeing, H., & Knüppel, S. (2019). Meal analysis for understanding eating behavior: meal- and participant-specific predictors for the variance in energy and macronutrient intake. *Nutrition journal*, *18*(1), 15. https://doi.org/10.1186/s12937-019-0440-8** |
| **118.**Antiglucocorticoid and related treatments for psychosis | **Garner, B., Phillips, L. J., Bendall, S., & Hetrick, S. E. (2016). Antiglucocorticoid and related treatments for psychosis. *The Cochrane database of systematic reviews*, *2016*(1), CD006995. https://doi.org/10.1002/14651858.CD006995.pub2** |
| **119.**Common genetic variation in obesity, lipid transfer genes and risk of Metabolic Syndrome: Results from IDEFICS/I.Family study and meta-analysis | **Nagrani, R., Foraita, R., Gianfagna, F., Iacoviello, L., Marild, S., Michels, N., Molnár, D., Moreno, L., Russo, P., Veidebaum, T., Ahrens, W., & Marron, M. (2020). Common genetic variation in obesity, lipid transfer genes and risk of Metabolic Syndrome: Results from IDEFICS/I.Family study and meta-analysis. *Scientific reports*, *10*(1), 7189. https://doi.org/10.1038/s41598-020-64031-2** |
| **120.**Meta-analysis of clinical metabolic profiling studies in cancer: challenges and opportunities | **Goveia, J., Pircher, A., Conradi, L. C., Kalucka, J., Lagani, V., Dewerchin, M., Eelen, G., DeBerardinis, R. J., Wilson, I. D., & Carmeliet, P. (2016). Meta-analysis of clinical metabolic profiling studies in cancer: challenges and opportunities. *EMBO molecular medicine*, *8*(10), 1134–1142.** [**https://doi.org/10.15252/emmm.201606798**](https://doi.org/10.15252/emmm.201606798) |
| **121.**Maternal BMI is positively associated with human milk fat: a systematic review and meta-regression analysis | **Daniel, A. I., Shama, S., Ismail, S., Bourdon, C., Kiss, A., Mwangome, M., Bandsma, R. H. J., & O'Connor, D. L. (2021). Maternal BMI is positively associated with human milk fat: a systematic review and meta-regression analysis. *The American journal of clinical nutrition*, *113*(4), 1009–1022. https://doi.org/10.1093/ajcn/nqaa410** |
| **122.**Adverse events during oral colchicine use: a systematic review and meta-analysis of randomised controlled trials | **Stewart, S., Yang, K. C. K., Atkins, K., Dalbeth, N., & Robinson, P. C. (2020). Adverse events during oral colchicine use: a systematic review and meta-analysis of randomised controlled trials. *Arthritis research & therapy*, *22*(1), 28. https://doi.org/10.1186/s13075-020-2120-7** |
| **123.**Influences of Ketogenic Diet on Body Fat Percentage, Respiratory Exchange Rate, and Total Cholesterol in Athletes: A Systematic Review and Meta-Analysis | **Lee, H. S., & Lee, J. (2021). Influences of Ketogenic Diet on Body Fat Percentage, Respiratory Exchange Rate, and Total Cholesterol in Athletes: A Systematic Review and Meta-Analysis. *International journal of environmental research and public health*, *18*(6), 2912. https://doi.org/10.3390/ijerph18062912** |
| **124.**The efficacy and safety of SGLT2 inhibitors for adjunctive treatment of type 1 diabetes: a systematic review and meta-analysis | **Chen, J., Fan, F., Wang, J. Y., Long, Y., Gao, C. L., Stanton, R. C., & Xu, Y. (2017). The efficacy and safety of SGLT2 inhibitors for adjunctive treatment of type 1 diabetes: a systematic review and meta-analysis. *Scientific reports*, *7*, 44128. https://doi.org/10.1038/srep44128** |
| **125.**Topical non-steroidal anti-inflammatory drugs for analgesia in traumatic corneal abrasions | **Wakai, A., Lawrenson, J. G., Lawrenson, A. L., Wang, Y., Brown, M. D., Quirke, M., Ghandour, O., McCormick, R., Walsh, C. D., Amayem, A., Lang, E., & Harrison, N. (2017). Topical non-steroidal anti-inflammatory drugs for analgesia in traumatic corneal abrasions. *The Cochrane database of systematic reviews*, *5*(5), CD009781. https://doi.org/10.1002/14651858.CD009781.pub2** |
| **126.**Modeling fatty acids for dairy cattle: Models to predict total fatty acid concentration and fatty acid digestion of feedstuffs | **Daley, V. L., Armentano, L. E., Kononoff, P. J., & Hanigan, M. D. (2020). Modeling fatty acids for dairy cattle: Models to predict total fatty acid concentration and fatty acid digestion of feedstuffs. *Journal of dairy science*, *103*(8), 6982–6999. https://doi.org/10.3168/jds.2019-17407** |
| **127.**Influence of fat and carbohydrate proportions on the metabolic profile in patients with type 2 diabetes: a meta-analysis | **Kodama, S., Saito, K., Tanaka, S., Maki, M., Yachi, Y., Sato, M., Sugawara, A., Totsuka, K., Shimano, H., Ohashi, Y., Yamada, N., & Sone, H. (2009). Influence of fat and carbohydrate proportions on the metabolic profile in patients with type 2 diabetes: a meta-analysis. *Diabetes care*, *32*(5), 959–965. https://doi.org/10.2337/dc08-1716** |
| **128.**A meta-analysis and meta-regression of the effect of forage particle size, level, source, and preservation method on feed intake, nutrient digestibility, and performance in dairy cows | **Nasrollahi, S. M., Imani, M., & Zebeli, Q. (2015). A meta-analysis and meta-regression of the effect of forage particle size, level, source, and preservation method on feed intake, nutrient digestibility, and performance in dairy cows. *Journal of dairy science*, *98*(12), 8926–8939. https://doi.org/10.3168/jds.2015-9681** |
| **129.**G1359A Variant of the Cannabinoid Receptor Gene (rs1049353) and Obesity-Related Traits and Related Endophenotypes: A Meta-Analysis | **Sadeghian, M., Rahmani, S., & Mansoori, A. (2018). G1359A Variant of the Cannabinoid Receptor Gene (rs1049353) and Obesity-Related Traits and Related Endophenotypes: A Meta-Analysis. *Annals of nutrition & metabolism*, *73*(1), 76–85. https://doi.org/10.1159/000490668** |
| **130.**The Effect of a Ketogenic Low-Carbohydrate, High-Fat Diet on Aerobic Capacity and Exercise Performance in Endurance Athletes: A Systematic Review and Meta-Analysis | **Cao, J., Lei, S., Wang, X., & Cheng, S. (2021). The Effect of a Ketogenic Low-Carbohydrate, High-Fat Diet on Aerobic Capacity and Exercise Performance in Endurance Athletes: A Systematic Review and Meta-Analysis. *Nutrients*, *13*(8), 2896. https://doi.org/10.3390/nu13082896** |
| **131.**Dietary behaviours in the context of nutrition transition: a systematic review and meta-analyses in two African countries | **Rousham, E. K., Pradeilles, R., Akparibo, R., Aryeetey, R., Bash, K., Booth, A., Muthuri, S. K., Osei-Kwasi, H., Marr, C. M., Norris, T., & Holdsworth, M. (2020). Dietary behaviours in the context of nutrition transition: a systematic review and meta-analyses in two African countries. *Public health nutrition*, *23*(11), 1948–1964. https://doi.org/10.1017/S1368980019004014** |
| **132.**Impact of FTO genotypes on BMI and weight in polycystic ovary syndrome: a systematic review and meta-analysis | **Wojciechowski, P., Lipowska, A., Rys, P., Ewens, K. G., Franks, S., Tan, S., Lerchbaum, E., Vcelak, J., Attaoua, R., Straczkowski, M., Azziz, R., Barber, T. M., Hinney, A., Obermayer-Pietsch, B., Lukasova, P., Bendlova, B., Grigorescu, F., Kowalska, I., Goodarzi, M. O., GIANT Consortium, … Malecki, M. T. (2012). Impact of FTO genotypes on BMI and weight in polycystic ovary syndrome: a systematic review and meta-analysis. *Diabetologia*, *55*(10), 2636–2645. https://doi.org/10.1007/s00125-012-2638-6** |
| **133.**Gene-Environment Interactions of Circadian-Related Genes for Cardiometabolic Traits | **Dashti, H. S., Follis, J. L., Smith, C. E., Tanaka, T., Garaulet, M., Gottlieb, D. J., Hruby, A., Jacques, P. F., Kiefte-de Jong, J. C., Lamon-Fava, S., Scheer, F. A., Bartz, T. M., Kovanen, L., Wojczynski, M. K., Frazier-Wood, A. C., Ahluwalia, T. S., Perälä, M. M., Jonsson, A., Muka, T., Kalafati, I. P., … CHARGE Nutrition Study Group (2015). Gene-Environment Interactions of Circadian-Related Genes for Cardiometabolic Traits. *Diabetes care*, *38*(8), 1456–1466. https://doi.org/10.2337/dc14-2709** |
| **134.**Adiposity and risk of decline in glomerular filtration rate: meta-analysis of individual participant data in a global consortium | **Chang, A. R., Grams, M. E., Ballew, S. H., Bilo, H., Correa, A., Evans, M., Gutierrez, O. M., Hosseinpanah, F., Iseki, K., Kenealy, T., Klein, B., Kronenberg, F., Lee, B. J., Li, Y., Miura, K., Navaneethan, S. D., Roderick, P. J., Valdivielso, J. M., Visseren, F. L. J., Zhang, L., … CKD Prognosis Consortium (CKD-PC) (2019). Adiposity and risk of decline in glomerular filtration rate: meta-analysis of individual participant data in a global consortium. BMJ (Clinical research ed.), 364, k5301. https://doi.org/10.1136/bmj.k5301** |
| **135.**Safety of oral use of nimesulide in children: systematic review of randomized controlled trials | **Gupta, P., & Sachdev, H. P. (2003). Safety of oral use of nimesulide in children: systematic review of randomized controlled trials. Indian pediatrics, 40(6), 518–531.** |
| **136.**Association between fat mass- and obesity-associated (FTO) gene polymorphism and polycystic ovary syndrome: a meta-analysis | **Cai, X., Liu, C., & Mou, S. (2014). Association between fat mass- and obesity-associated (FTO) gene polymorphism and polycystic ovary syndrome: a meta-analysis. PloS one, 9(1), e86972. https://doi.org/10.1371/journal.pone.0086972** |
| **137.**Differential gene expression in dairy cows under negative energy balance and ketosis: A systematic review and meta-analysis | **Soares, R. A. N., Vargas, G., Muniz, M. M. M., Soares, M. A. M., Cánovas, A., Schenkel, F., & Squires, E. J. (2021). Differential gene expression in dairy cows under negative energy balance and ketosis: A systematic review and meta-analysis. Journal of dairy science, 104(1), 602–615. https://doi.org/10.3168/jds.2020-18883** |
| **138.**Diseases, reproductive performance, and changes in milk production associated with subclinical ketosis in dairy cows: a meta-analysis and review | **Raboisson, D., Mounié, M., & Maigné, E. (2014). Diseases, reproductive performance, and changes in milk production associated with subclinical ketosis in dairy cows: a meta-analysis and review. Journal of dairy science, 97(12), 7547–7563. https://doi.org/10.3168/jds.2014-8237** |
| **139.**Telithromycin in the treatment of pneumococcal community-acquired respiratory tract infections: a review | **Fogarty, C. M., Buchanan, P., Aubier, M., Baz, M., van Rensburg, D., Rangaraju, M., & Nusrat, R. (2006). Telithromycin in the treatment of pneumococcal community-acquired respiratory tract infections: a review. International journal of infectious diseases : IJID : official publication of the International Society for Infectious Diseases, 10(2), 136–147. https://doi.org/10.1016/j.ijid.2005.01.003** |
| **140.**The relative efficacy of meperidine for the treatment of acute migraine: a meta-analysis of randomized controlled trials | **Friedman, B. W., Kapoor, A., Friedman, M. S., Hochberg, M. L., & Rowe, B. H. (2008). The relative efficacy of meperidine for the treatment of acute migraine: a meta-analysis of randomized controlled trials. Annals of emergency medicine, 52(6), 705–713. https://doi.org/10.1016/j.annemergmed.2008.05.036** |
| **141.**The Association between High Fat Diet around Gestation and Metabolic Syndrome-related Phenotypes in Rats: A Systematic Review and Meta-Analysis | **Tellechea, M. L., Mensegue, M. F., & Pirola, C. J. (2017). The Association between High Fat Diet around Gestation and Metabolic Syndrome-related Phenotypes in Rats: A Systematic Review and Meta-Analysis. Scientific reports, 7(1), 5086. https://doi.org/10.1038/s41598-017-05344-7** |
| **142.**Compared efficacy of preservation solutions on the outcome of liver transplantation: Meta-analysis | **Szilágyi, Á. L., Mátrai, P., Hegyi, P., Tuboly, E., Pécz, D., Garami, A., Solymár, M., Pétervári, E., Balaskó, M., Veres, G., Czopf, L., Wobbe, B., Szabó, D., Wagner, J., & Hartmann, P. (2018). Compared efficacy of preservation solutions on the outcome of liver transplantation: Meta-analysis. World journal of gastroenterology, 24(16), 1812–1824. https://doi.org/10.3748/wjg.v24.i16.1812** |
| **143.**The Effect of Dietary Fibre on Gut Microbiota, Lipid Profile, and Inflammatory Markers in Patients with Type 2 Diabetes: A Systematic Review and Meta-Analysis of Randomised Controlled Trials | **Ojo, O., Ojo, O. O., Zand, N., & Wang, X. (2021). The Effect of Dietary Fibre on Gut Microbiota, Lipid Profile, and Inflammatory Markers in Patients with Type 2 Diabetes: A Systematic Review and Meta-Analysis of Randomised Controlled Trials. Nutrients, 13(6), 1805. https://doi.org/10.3390/nu13061805** |
| **144.**Timing of Debridement and Infection Rates in Open Fractures of the Hand: A Systematic Review | **Ketonis, C., Dwyer, J., & Ilyas, A. M. (2017). Timing of Debridement and Infection Rates in Open Fractures of the Hand: A Systematic Review. Hand (New York, N.Y.), 12(2), 119–126. https://doi.org/10.1177/1558944716643294** |
| **145.**A meta-analysis of feed digestion in dairy cows. 2. The effects of feeding level and diet composition on digestibility | **Huhtanen, P., Rinne, M., & Nousiainen, J. (2009). A meta-analysis of feed digestion in dairy cows. 2. The effects of feeding level and diet composition on digestibility. Journal of dairy science, 92(10), 5031–5042. https://doi.org/10.3168/jds.2008-1834** |
| **146.**Meta-analysis of sequence-based association studies across three cattle breeds reveals 25 QTL for fat and protein percentages in milk at nucleotide resolution | **Pausch, H., Emmerling, R., Gredler-Grandl, B., Fries, R., Daetwyler, H. D., & Goddard, M. E. (2017). Meta-analysis of sequence-based association studies across three cattle breeds reveals 25 QTL for fat and protein percentages in milk at nucleotide resolution. BMC genomics, 18(1), 853. https://doi.org/10.1186/s12864-017-4263-8** |
| **147.**Ibuprofen for acute treatment of episodic tension-type headache in adults | **Derry, S., Wiffen, P. J., Moore, R. A., & Bendtsen, L. (2015). Ibuprofen for acute treatment of episodic tension-type headache in adults. The Cochrane database of systematic reviews, 2015(7), CD011474. https://doi.org/10.1002/14651858.CD011474.pub2** |
| **148.**Genome-wide meta-analysis of observational studies shows common genetic variants associated with macronutrient intake | **Tanaka, T., Ngwa, J. S., van Rooij, F. J., Zillikens, M. C., Wojczynski, M. K., Frazier-Wood, A. C., Houston, D. K., Kanoni, S., Lemaitre, R. N., Luan, J., Mikkilä, V., Renstrom, F., Sonestedt, E., Zhao, J. H., Chu, A. Y., Qi, L., Chasman, D. I., de Oliveira Otto, M. C., Dhurandhar, E. J., Feitosa, M. F., … Nettleton, J. A. (2013). Genome-wide meta-analysis of observational studies shows common genetic variants associated with macronutrient intake. The American journal of clinical nutrition, 97(6), 1395–1402. https://doi.org/10.3945/ajcn.112.052183** |
| **149.**Effect of magnesium sulfate on renal colic pain: A PRISMA-compliant meta-analysis | **Chen, L. F., Yang, C. H., Lin, T. Y., Pao, P. J., Chu, K. C., Hsu, C. W., Bai, C. H., Du, M. H., & Hsu, Y. P. (2020). Effect of magnesium sulfate on renal colic pain: A PRISMA-compliant meta-analysis. Medicine, 99(46), e23279. https://doi.org/10.1097/MD.0000000000023279** |
| **150.**Feed intake and milk production in dairy cows fed different grass and legume species: a meta-analysis | **Johansen, M., Lund, P., & Weisbjerg, M. R. (2018). Feed intake and milk production in dairy cows fed different grass and legume species: a meta-analysis. Animal : an international journal of animal bioscience, 12(1), 66–75. https://doi.org/10.1017/S1751731117001215** |
| **151.**A Bayesian meta-analysis on published sample mean and variance pharmacokinetic data with application to drug-drug interaction prediction | **Yu, M., Kim, S., Wang, Z., Hall, S., & Li, L. (2008). A Bayesian meta-analysis on published sample mean and variance pharmacokinetic data with application to drug-drug interaction prediction. Journal of biopharmaceutical statistics, 18(6), 1063–1083. https://doi.org/10.1080/10543400802369004** |
| **152.**A multivariate meta-analysis of motivational interviewing process and outcome | **Yu, M., Kim, S., Wang, Z., Hall, S., & Li, L. (2008). A Bayesian meta-analysis on published sample mean and variance pharmacokinetic data with application to drug-drug interaction prediction. Journal of biopharmaceutical statistics, 18(6), 1063–1083. https://doi.org/10.1080/10543400802369004** |
| **153.**Influence of Ketorolac Supplementation on Pain Control for Knee Arthroscopy: A Meta-Analysis of Randomized Controlled Trials | **Wan, R. J., Liu, S. F., Kuang, Z. P., Ran, Q., Zhao, C., & Huang, W. (2020). Influence of Ketorolac Supplementation on Pain Control for Knee Arthroscopy: A Meta-Analysis of Randomized Controlled Trials. Orthopaedic surgery, 12(1), 31–37. https://doi.org/10.1111/os.12608** |
| **154.**Interventions for treating oral candidiasis for patients with cancer receiving treatment | **Worthington, H. V., Clarkson, J. E., & Eden, O. B. (2007). Interventions for treating oral candidiasis for patients with cancer receiving treatment. The Cochrane database of systematic reviews, (2), CD001972. https://doi.org/10.1002/14651858.CD001972.pub3** |
| **155.**Outcomes in idiopathic pulmonary fibrosis: a meta-analysis from placebo controlled trials | **Atkins, C. P., Loke, Y. K., & Wilson, A. M. (2014). Outcomes in idiopathic pulmonary fibrosis: a meta-analysis from placebo controlled trials. Respiratory medicine, 108(2), 376–387. https://doi.org/10.1016/j.rmed.2013.11.007** |
| **156.**Oral treatments for fungal infections of the skin of the foot | **Bell-Syer, S. E., Hart, R., Crawford, F., Torgerson, D. J., Tyrrell, W., & Russell, I. (2002). Oral treatments for fungal infections of the skin of the foot. The Cochrane database of systematic reviews, (2), CD003584. https://doi.org/10.1002/14651858.CD003584** |
| **157.**Critical review of health effects of soyabean phyto-oestrogens in post-menopausal women | **Cassidy, A., Albertazzi, P., Lise Nielsen, I., Hall, W., Williamson, G., Tetens, I., Atkins, S., Cross, H., Manios, Y., Wolk, A., Steiner, C., & Branca, F. (2006). Critical review of health effects of soyabean phyto-oestrogens in post-menopausal women. The Proceedings of the Nutrition Society, 65(1), 76–92. https://doi.org/10.1079/pns2005476** |
| **158.**Effects of Sodium-Glucose Cotransporter Inhibitor/Glucagon-Like Peptide-1 Receptor Agonist Add-On to Insulin Therapy on Glucose Homeostasis and Body Weight in Patients With Type 1 Diabetes: A Network Meta-Analysis | **Kim, Y. J., Hwang, S. D., & Lim, S. (2020). Effects of Sodium-Glucose Cotransporter Inhibitor/Glucagon-Like Peptide-1 Receptor Agonist Add-On to Insulin Therapy on Glucose Homeostasis and Body Weight in Patients With Type 1 Diabetes: A Network Meta-Analysis. Frontiers in endocrinology, 11, 553. https://doi.org/10.3389/fendo.2020.00553** |
| **159.**Single dose dipyrone for acute postoperative pain | **Edwards, J., Meseguer, F., Faura, C., Moore, R. A., McQuay, H. J., & Derry, S. (2010). Single dose dipyrone for acute postoperative pain. The Cochrane database of systematic reviews, (9), CD003227. https://doi.org/10.1002/14651858.CD003227.pub2** |
| **160.**Effects of high and low fat dairy food on cardio-metabolic risk factors: a meta-analysis of randomized studies | **Benatar, J. R., Sidhu, K., & Stewart, R. A. (2013). Effects of high and low fat dairy food on cardio-metabolic risk factors: a meta-analysis of randomized studies. PloS one, 8(10), e76480. https://doi.org/10.1371/journal.pone.0076480** |
| **161.**Effects of monounsaturated fatty acids on glycaemic control in patients with abnormal glucose metabolism: a systematic review and meta-analysis | **Schwingshackl, L., Strasser, B., & Hoffmann, G. (2011). Effects of monounsaturated fatty acids on glycaemic control in patients with abnormal glucose metabolism: a systematic review and meta-analysis. Annals of nutrition & metabolism, 58(4), 290–296. https://doi.org/10.1159/000331214** |
| **162.**Replication of FTO Gene associated with lean mass in a Meta-Analysis of Genome-Wide Association Studies | **Ran, S., Jiang, Z. X., He, X., Liu, Y., Zhang, Y. X., Zhang, L., Pei, Y. F., Zhang, M., Hai, R., Gu, G. S., Liu, B. L., Tian, Q., Zhang, Y. H., Wang, J. Y., & Deng, H. W. (2020). Replication of FTO Gene associated with lean mass in a Meta-Analysis of Genome-Wide Association Studies. Scientific reports, 10(1), 5057. https://doi.org/10.1038/s41598-020-61406-3** |
| **163.**Efficacy of Eribulin mesylate in older patients with breast cancer: A pooled analysis of clinical trial and real-world data | **Pedersini, R., di Mauro, P., Amoroso, V., Parati, M. C., Turla, A., Ghilardi, M., Vassalli, L., Ardine, M., Volta, A. D., Monteverdi, S., Borgonovo, K., Ghidini, A., Cabiddu, M., Simoncini, E. L., Petrelli, F., Berruti, A., & Barni, S. (2020). Efficacy of Eribulin mesylate in older patients with breast cancer: A pooled analysis of clinical trial and real-world data. Journal of geriatric oncology, 11(6), 976–981. https://doi.org/10.1016/j.jgo.2020.03.021** |
| **164.**Short communication: Prediction of intake in dairy cows under tropical conditions | **Souza, M. C., Oliveira, A. S., Araújo, C. V., Brito, A. F., Teixeira, R. M., Moares, E. H., & Moura, D. C. (2014). Short communication: Prediction of intake in dairy cows under tropical conditions. Journal of dairy science, 97(6), 3845–3854. https://doi.org/10.3168/jds.2013-7652** |
| **165.**Routine versus selective antifungal administration for control of fungal infections in patients with cancer | **Gotzsche, P. C., & Johansen, H. K. (2002). Routine versus selective antifungal administration for control of fungal infections in patients with cancer. The Cochrane database of systematic reviews, (2), CD000026. https://doi.org/10.1002/14651858.CD000026** |
| **166.**Association of fat-mass and obesity-associated gene FTO rs9939609 polymorphism with the risk of obesity among children and adolescents: a meta-analysis | **Quan, L. L., Wang, H., Tian, Y., Mu, X., Zhang, Y., & Tao, K. (2015). Association of fat-mass and obesity-associated gene FTO rs9939609 polymorphism with the risk of obesity among children and adolescents: a meta-analysis. European review for medical and pharmacological sciences, 19(4), 614–623.** |
| **167.**Meta-analysis added power to identify variants in FTO associated with type 2 diabetes and obesity in the Asian population | **Liu, Y., Liu, Z., Song, Y., Zhou, D., Zhang, D., Zhao, T., Chen, Z., Yu, L., Yang, Y., Feng, G., Li, J., Zhang, J., Liu, S., Zhang, Z., He, L., & Xu, H. (2010). Meta-analysis added power to identify variants in FTO associated with type 2 diabetes and obesity in the Asian population. Obesity (Silver Spring, Md.), 18(8), 1619–1624. https://doi.org/10.1038/oby.2009.469** |
| **168.**A meta-analysis on the effect of dietary application of exogenous fibrolytic enzymes on the performance of dairy cows | **Arriola, K. G., Oliveira, A. S., Ma, Z. X., Lean, I. J., Giurcanu, M. C., & Adesogan, A. T. (2017). A meta-analysis on the effect of dietary application of exogenous fibrolytic enzymes on the performance of dairy cows. Journal of dairy science, 100(6), 4513–4527. https://doi.org/10.3168/jds.2016-12103** |
| **169.**Therapeutic Options for Patients with Refractory Status Epilepticus in Palliative Settings or with a Limitation of Life-Sustaining Therapies: A Systematic Review | **Willems, L. M., Bauer, S., Jahnke, K., Voss, M., Rosenow, F., & Strzelczyk, A. (2020). Therapeutic Options for Patients with Refractory Status Epilepticus in Palliative Settings or with a Limitation of Life-Sustaining Therapies: A Systematic Review. CNS drugs, 34(8), 801–826. https://doi.org/10.1007/s40263-020-00747-z** |
| **170.**Safety of Ramadan fasting in young patients with type 1 diabetes: A systematic review and meta-analysis | **Loh, H. H., Lim, L. L., Loh, H. S., & Yee, A. (2019). Safety of Ramadan fasting in young patients with type 1 diabetes: A systematic review and meta-analysis. Journal of diabetes investigation, 10(6), 1490–1501. https://doi.org/10.1111/jdi.13054** |
| **171.**[β-hydroxy-β-methylbutyrate as a dietary supplement (I): metabolism and toxicity] | **Manjarrez-Montes-de-Oca, R., Torres-Vaca, M., González-Gallego, J., & Alvear-Ordenes, I. (2014). El β-hidroxi-β-metilbutirato (HMB) como suplemento nutricional (I): metabolismo y toxicidad [β-hydroxy-β-methylbutyrate as a dietary supplement (I): metabolism and toxicity]. Nutricion hospitalaria, 31(2), 590–596. https://doi.org/10.3305/nh.2015.31.2.8432** |
| **172.**A meta-analysis of the impact of monensin in lactating dairy cattle. Part 3. Health and reproduction | **Duffield, T. F., Rabiee, A. R., & Lean, I. J. (2008). A meta-analysis of the impact of monensin in lactating dairy cattle. Part 3. Health and reproduction. Journal of dairy science, 91(6), 2328–2341. https://doi.org/10.3168/jds.2007-0801** |
| **173.**Ketosis proportionately spares glucose utilization in brain | **Zhang, Y., Kuang, Y., Xu, K., Harris, D., Lee, Z., LaManna, J., & Puchowicz, M. A. (2013). Ketosis proportionately spares glucose utilization in brain. *Journal of cerebral blood flow and metabolism : official journal of the International Society of Cerebral Blood Flow and Metabolism*, *33*(8), 1307–1311. https://doi.org/10.1038/jcbfm.2013.87** |
| **174.**Empiric antibiotic coverage of atypical pathogens for community-acquired pneumonia in hospitalized adults | **Robenshtok, E., Shefet, D., Gafter-Gvili, A., Paul, M., Vidal, L., & Leibovici, L. (2008). Empiric antibiotic coverage of atypical pathogens for community acquired pneumonia in hospitalized adults. *The Cochrane database of systematic reviews*, (1), CD004418. https://doi.org/10.1002/14651858.CD004418.pub3** |
| **175.**The efficacy of dexketoprofen for migraine attack: A meta-analysis of randomized controlled studies | **Yang, B., Xu, Z., Chen, L., Chen, X., & Xie, Y. (2019). The efficacy of dexketoprofen for migraine attack: A meta-analysis of randomized controlled studies. *Medicine*, *98*(46), e17734. https://doi.org/10.1097/MD.0000000000017734** |
| **176.**Empirical prediction of net splanchnic release of ketogenic nutrients, acetate, butyrate and β-hydroxybutyrate in ruminants: a meta-analysis | **Loncke, C., Nozière, P., Bahloul, L., Vernet, J., Lapierre, H., Sauvant, D., & Ortigues-Marty, I. (2015). Empirical prediction of net splanchnic release of ketogenic nutrients, acetate, butyrate and β-hydroxybutyrate in ruminants: a meta-analysis. *Animal : an international journal of animal bioscience*, *9*(3), 449–463. https://doi.org/10.1017/S1751731114002638** |
| **177.**Novel locus including FGF21 is associated with dietary macronutrient intake | **Chu, A. Y., Workalemahu, T., Paynter, N. P., Rose, L. M., Giulianini, F., Tanaka, T., Ngwa, J. S., CHARGE Nutrition Working Group, Qi, Q., Curhan, G. C., Rimm, E. B., Hunter, D. J., Pasquale, L. R., Ridker, P. M., Hu, F. B., Chasman, D. I., Qi, L., & DietGen Consortium (2013). Novel locus including FGF21 is associated with dietary macronutrient intake. *Human molecular genetics*, *22*(9), 1895–1902. https://doi.org/10.1093/hmg/ddt032** |
| **178.**Relationship of Estimated GFR and Albuminuria to Concurrent Laboratory Abnormalities: An Individual Participant Data Meta-analysis in a Global Consortium | **Inker, L. A., Grams, M. E., Levey, A. S., Coresh, J., Cirillo, M., Collins, J. F., Gansevoort, R. T., Gutierrez, O. M., Hamano, T., Heine, G. H., Ishikawa, S., Jee, S. H., Kronenberg, F., Landray, M. J., Miura, K., Nadkarni, G. N., Peralta, C. A., Rothenbacher, D., Schaeffner, E., Sedaghat, S., … CKD Prognosis Consortium (2019). Relationship of Estimated GFR and Albuminuria to Concurrent Laboratory Abnormalities: An Individual Participant Data Meta-analysis in a Global Consortium. *American journal of kidney diseases : the official journal of the National Kidney Foundation*, *73*(2), 206–217. https://doi.org/10.1053/j.ajkd.2018.08.013** |
| **179.**Effects of feeding extruded linseed on production performance and milk fatty acid profile in dairy cows: A meta-analysis | **Meignan, T., Lechartier, C., Chesneau, G., & Bareille, N. (2017). Effects of feeding extruded linseed on production performance and milk fatty acid profile in dairy cows: A meta-analysis. *Journal of dairy science*, *100*(6), 4394–4408. https://doi.org/10.3168/jds.2016-11850** |
| **180.**Energy restriction and the risk of spontaneous mammary tumors in mice: a meta-analysis | **Dirx, M. J., Zeegers, M. P., Dagnelie, P. C., van den Bogaard, T., & van den Brandt, P. A. (2003). Energy restriction and the risk of spontaneous mammary tumors in mice: a meta-analysis. *International journal of cancer*, *106*(5), 766–770. https://doi.org/10.1002/ijc.11277** |
| **181.**Is PEEK cage better than titanium cage in anterior cervical discectomy and fusion surgery? A meta-analysis | **Li, Z. J., Wang, Y., Xu, G. J., & Tian, P. (2016). Is PEEK cage better than titanium cage in anterior cervical discectomy and fusion surgery? A meta-analysis. *BMC musculoskeletal disorders*, *17*(1), 379. https://doi.org/10.1186/s12891-016-1234-1** |
| **182.**A meta-analysis of the impact of monensin in lactating dairy cattle. Part 1. Metabolic effects | **Duffield, T. F., Rabiee, A. R., & Lean, I. J. (2008). A meta-analysis of the impact of monensin in lactating dairy cattle. Part 1. Metabolic effects. *Journal of dairy science*, *91*(4), 1334–1346. https://doi.org/10.3168/jds.2007-0607** |
| **183.**Preservation solutions for static cold storage of kidney allografts: a systematic review and meta-analysis | **O'Callaghan, J. M., Knight, S. R., Morgan, R. D., & Morris, P. J. (2012). Preservation solutions for static cold storage of kidney allografts: a systematic review and meta-analysis. *American journal of transplantation : official journal of the American Society of Transplantation and the American Society of Transplant Surgeons*, *12*(4), 896–906. https://doi.org/10.1111/j.1600-6143.2011.03908.x** |
| **184.**Single dose oral ketoprofen and dexketoprofen for acute postoperative pain in adults | **Barden, J., Derry, S., McQuay, H. J., & Moore, R. A. (2009). Single dose oral ketoprofen and dexketoprofen for acute postoperative pain in adults. *The Cochrane database of systematic reviews*, (4), CD007355. https://doi.org/10.1002/14651858.CD007355.pub2** |
| **185.**Targeting intensive versus conventional glycaemic control for type 1 diabetes mellitus: a systematic review with meta-analyses and trial sequential analyses of randomised clinical trials | **Kähler, P., Grevstad, B., Almdal, T., Gluud, C., Wetterslev, J., Lund, S. S., Vaag, A., & Hemmingsen, B. (2014). Targeting intensive versus conventional glycaemic control for type 1 diabetes mellitus: a systematic review with meta-analyses and trial sequential analyses of randomised clinical trials. *BMJ open*, *4*(8), e004806. https://doi.org/10.1136/bmjopen-2014-004806** |
| **186.**Systematic review of preservation solutions for allografts for liver transplantation based on a network meta-analysis | **Rao, F., Yang, J., Gong, C., Huang, R., Wang, Q., & Shen, J. (2018). Systematic review of preservation solutions for allografts for liver transplantation based on a network meta-analysis. *International journal of surgery (London, England)*, *54*(Pt A), 1–6. https://doi.org/10.1016/j.ijsu.2018.04.024** |
| **187.**Efficacy and safety of chimeric antigen receptor T-cell (CAR-T) therapy in patients with haematological and solid malignancies: protocol for a systematic review and meta-analysis | **Grigor, E. J. M., Fergusson, D. A., Haggar, F., Kekre, N., Atkins, H., Shorr, R., Holt, R. A., Hutton, B., Ramsay, T., Seftel, M., Jonker, D., Daugaard, M., Thavorn, K., Presseau, J., & Lalu, M. M. (2017). Efficacy and safety of chimeric antigen receptor T-cell (CAR-T) therapy in patients with haematological and solid malignancies: protocol for a systematic review and meta-analysis. *BMJ open*, *7*(12), e019321. https://doi.org/10.1136/bmjopen-2017-019321** |
| **188.**Interventions for the prevention and management of oropharyngeal candidiasis associated with HIV infection in adults and children | **Pienaar, E. D., Young, T., & Holmes, H. (2010). Interventions for the prevention and management of oropharyngeal candidiasis associated with HIV infection in adults and children. *The Cochrane database of systematic reviews*, *2010*(11), CD003940. https://doi.org/10.1002/14651858.CD003940.pub3** |
| **189.**Cardiovascular outcomes associated with SGLT-2 inhibitors versus other glucose-lowering drugs in patients with type 2 diabetes: A real-world systematic review and meta-analysis | **Li, C. X., Liang, S., Gao, L., & Liu, H. (2021). Cardiovascular outcomes associated with SGLT-2 inhibitors versus other glucose-lowering drugs in patients with type 2 diabetes: A real-world systematic review and meta-analysis. *PloS one*, *16*(2), e0244689. https://doi.org/10.1371/journal.pone.0244689** |
| **190.**Project INTEGRATE: An integrative study of brief alcohol interventions for college students | **Mun, E. Y., de la Torre, J., Atkins, D. C., White, H. R., Ray, A. E., Kim, S. Y., Jiao, Y., Clarke, N., Huo, Y., Larimer, M. E., Huh, D., & Project INTEGRATE Team (2015). Project INTEGRATE: An integrative study of brief alcohol interventions for college students. *Psychology of addictive behaviors : journal of the Society of Psychologists in Addictive Behaviors*, *29*(1), 34–48. https://doi.org/10.1037/adb0000047** |
| **191.**Comparison of the efficacy and patients' tolerability of Nepafenac and Ketorolac in the treatment of ocular inflammation following cataract surgery: A meta-analysis of randomized controlled trials | **Zhao, X., Xia, S., Wang, E., & Chen, Y. (2017). Comparison of the efficacy and patients' tolerability of Nepafenac and Ketorolac in the treatment of ocular inflammation following cataract surgery: A meta-analysis of randomized controlled trials. *PloS one*, *12*(3), e0173254. https://doi.org/10.1371/journal.pone.0173254** |
| **192.**A meta-analysis review of the effects of recombinant bovine somatotropin. 2. Effects on animal health, reproductive performance, and culling | **Dohoo, I. R., DesCôteaux, L., Leslie, K., Fredeen, A., Shewfelt, W., Preston, A., & Dowling, P. (2003). A meta-analysis review of the effects of recombinant bovine somatotropin. 2. Effects on animal health, reproductive performance, and culling. *Canadian journal of veterinary research = Revue canadienne de recherche veterinaire*, *67*(4), 252–264.** |
| **193.**No effect of energy intake overall on risk of endometrial cancers: a meta-analysis | **Chu, K. T., Song, Y., & Zhou, J. H. (2014). No effect of energy intake overall on risk of endometrial cancers: a meta-analysis. *Asian Pacific journal of cancer prevention : APJCP*, *15*(23), 10293–10298. https://doi.org/10.7314/apjcp.2014.15.23.10293** |
| **194.**Pre-clinical animal models are poor predictors of human toxicities in phase 1 oncology clinical trials | **Atkins, J. T., George, G. C., Hess, K., Marcelo-Lewis, K. L., Yuan, Y., Borthakur, G., Khozin, S., LoRusso, P., & Hong, D. S. (2020). Pre-clinical animal models are poor predictors of human toxicities in phase 1 oncology clinical trials. *British journal of cancer*, *123*(10), 1496–1501. https://doi.org/10.1038/s41416-020-01033-x** |
| **195.**Safety and Tolerability of Empagliflozin in Patients with Type 2 Diabetes | **Kohler, S., Salsali, A., Hantel, S., Kaspers, S., Woerle, H. J., Kim, G., & Broedl, U. C. (2016). Safety and Tolerability of Empagliflozin in Patients with Type 2 Diabetes. *Clinical therapeutics*, *38*(6), 1299–1313. https://doi.org/10.1016/j.clinthera.2016.03.031** |
| **196.**WITHDRAWN: Single dose dipyrone for acute postoperative pain | **Derry, S., Faura, C., Edwards, J., McQuay, H. J., & Moore, R. A. (2013). WITHDRAWN: Single dose dipyrone for acute postoperative pain. *The Cochrane database of systematic reviews*, *2013*(11), CD003227. https://doi.org/10.1002/14651858.CD003227.pub3** |
| **197.**Oral xanthines as maintenance treatment for asthma in children | **Seddon, P., Bara, A., Ducharme, F. M., & Lasserson, T. J. (2006). Oral xanthines as maintenance treatment for asthma in children. *The Cochrane database of systematic reviews*, *2006*(1), CD002885. https://doi.org/10.1002/14651858.CD002885.pub2** |
| **198.**Genetic variants in FTO associated with metabolic syndrome: a meta- and gene-based analysis | **Wang, H., Dong, S., Xu, H., Qian, J., & Yang, J. (2012). Genetic variants in FTO associated with metabolic syndrome: a meta- and gene-based analysis. *Molecular biology reports*, *39*(5), 5691–5698. https://doi.org/10.1007/s11033-011-1377-y** |
| **199.**Clinical and bacteriologic efficacy of telithromycin in patients with bacteremic community-acquired pneumonia | **Carbon, C., van Rensburg, D., Hagberg, L., Fogarty, C., Tellier, G., Rangaraju, M., & Nusrat, R. (2006). Clinical and bacteriologic efficacy of telithromycin in patients with bacteremic community-acquired pneumonia. *Respiratory medicine*, *100*(4), 577–585. https://doi.org/10.1016/j.rmed.2005.11.007** |
| **200.**Comparison of the Efficacy of Glucagon-Like Peptide-1 Receptor Agonists in Patients With Metabolic Associated Fatty Liver Disease: Updated Systematic Review and Meta-Analysis | **Dai, Y., He, H., Li, S., Yang, L., Wang, X., Liu, Z., & An, Z. (2021). Comparison of the Efficacy of Glucagon-Like Peptide-1 Receptor Agonists in Patients With Metabolic Associated Fatty Liver Disease: Updated Systematic Review and Meta-Analysis. *Frontiers in endocrinology*, *11*, 622589. https://doi.org/10.3389/fendo.2020.622589** |
| **201.**Effectiveness of Oral Nutritional Supplements on Older People with Anorexia: A Systematic Review and Meta-Analysis of Randomized Controlled Trials | **Li, M., Zhao, S., Wu, S., Yang, X., & Feng, H. (2021). Effectiveness of Oral Nutritional Supplements on Older People with Anorexia: A Systematic Review and Meta-Analysis of Randomized Controlled Trials. *Nutrients*, *13*(3), 835. https://doi.org/10.3390/nu13030835** |
| **202.**The effectiveness of interventions aimed at increasing physical activity in adults with persistent musculoskeletal pain: a systematic review and meta-analysis | **Marley, J., Tully, M. A., Porter-Armstrong, A., Bunting, B., O'Hanlon, J., Atkins, L., Howes, S., & McDonough, S. M. (2017). The effectiveness of interventions aimed at increasing physical activity in adults with persistent musculoskeletal pain: a systematic review and meta-analysis. *BMC musculoskeletal disorders*, *18*(1), 482. https://doi.org/10.1186/s12891-017-1836-2** |
| **203.**Effects of nonsteroidal anti-inflammatory drugs on postoperative renal function in adults with normal renal function | **Lee, A., Cooper, M. G., Craig, J. C., Knight, J. F., & Keneally, J. P. (2007). Effects of nonsteroidal anti-inflammatory drugs on postoperative renal function in adults with normal renal function. *The Cochrane database of systematic reviews*, *2007*(2), CD002765. https://doi.org/10.1002/14651858.CD002765.pub3** |
| **204.**Implication of European-derived adiposity loci in African Americans | **Hester, J. M., Wing, M. R., Li, J., Palmer, N. D., Xu, J., Hicks, P. J., Roh, B. H., Norris, J. M., Wagenknecht, L. E., Langefeld, C. D., Freedman, B. I., Bowden, D. W., & Ng, M. C. (2012). Implication of European-derived adiposity loci in African Americans. *International journal of obesity (2005)*, *36*(3), 465–473. https://doi.org/10.1038/ijo.2011.131** |
| **205.**Distribution and Removal of Pharmaceuticals in Liquid and Solid Phases in the Unit Processes of Sewage Treatment Plants | **Park, J., Kim, C., Hong, Y., Lee, W., Chung, H., Jeong, D. H., & Kim, H. (2020). Distribution and Removal of Pharmaceuticals in Liquid and Solid Phases in the Unit Processes of Sewage Treatment Plants. *International journal of environmental research and public health*, *17*(3), 687. https://doi.org/10.3390/ijerph17030687** |
| **206.**FTO genetic variants and risk of obesity and type 2 diabetes: a meta-analysis of 28,394 Indians | **Vasan, S. K., Karpe, F., Gu, H. F., Brismar, K., Fall, C. H., Ingelsson, E., & Fall, T. (2014). FTO genetic variants and risk of obesity and type 2 diabetes: a meta-analysis of 28,394 Indians. *Obesity (Silver Spring, Md.)*, *22*(3), 964–970. https://doi.org/10.1002/oby.20606** |
| **207.**Comparison of risk prediction using the CKD-EPI equation and the MDRD study equation for estimated glomerular filtration rate | **Matsushita, K., Mahmoodi, B. K., Woodward, M., Emberson, J. R., Jafar, T. H., Jee, S. H., Polkinghorne, K. R., Shankar, A., Smith, D. H., Tonelli, M., Warnock, D. G., Wen, C. P., Coresh, J., Gansevoort, R. T., Hemmelgarn, B. R., Levey, A. S., & Chronic Kidney Disease Prognosis Consortium (2012). Comparison of risk prediction using the CKD-EPI equation and the MDRD study equation for estimated glomerular filtration rate. *JAMA*, *307*(18), 1941–1951. https://doi.org/10.1001/jama.2012.3954** |
| **208.**Patient-level pooled analysis of adjudicated gastrointestinal outcomes in celecoxib clinical trials: meta-analysis of 51,000 patients enrolled in 52 randomized trials | **Moore, A., Makinson, G., & Li, C. (2013). Patient-level pooled analysis of adjudicated gastrointestinal outcomes in celecoxib clinical trials: meta-analysis of 51,000 patients enrolled in 52 randomized trials. *Arthritis research & therapy*, *15*(1), R6. https://doi.org/10.1186/ar4134** |
| **209.**Meta-analysis of prophylactic or empirical antifungal treatment versus placebo or no treatment in patients with cancer complicated by neutropenia | **Gøtzsche, P. C., & Johansen, H. K. (1997). Meta-analysis of prophylactic or empirical antifungal treatment versus placebo or no treatment in patients with cancer complicated by neutropenia. *BMJ (Clinical research ed.)*, *314*(7089), 1238–1244. https://doi.org/10.1136/bmj.314.7089.1238** |
| **210.**FTO, type 2 diabetes, and weight gain throughout adult life: a meta-analysis of 41,504 subjects from the Scandinavian HUNT, MDC, and MPP studies | **Hertel, J. K., Johansson, S., Sonestedt, E., Jonsson, A., Lie, R. T., Platou, C. G., Nilsson, P. M., Rukh, G., Midthjell, K., Hveem, K., Melander, O., Groop, L., Lyssenko, V., Molven, A., Orho-Melander, M., & Njølstad, P. R. (2011). FTO, type 2 diabetes, and weight gain throughout adult life: a meta-analysis of 41,504 subjects from the Scandinavian HUNT, MDC, and MPP studies. *Diabetes*, *60*(5), 1637–1644. https://doi.org/10.2337/db10-1340** |
| **211.**[Impact of glucagon-like peptide-1 receptor agonists on nasopharyngitis and upper respiratory tract infection among patients with type 2 diabetes: a network meta-analysis] | **Li, Z. X., Wu, S. S., Yang, Z. R., Zhan, S. Y., & Sun, F. (2016). *Beijing da xue xue bao. Yi xue ban = Journal of Peking University. Health sciences*, *48*(3), 454–459.** |
| **212.**High volume local infiltration analgesia compared to peripheral nerve block for hip and knee arthroplasty-what is the evidence? | **Fowler, S. J., & Christelis, N. (2013). High volume local infiltration analgesia compared to peripheral nerve block for hip and knee arthroplasty-what is the evidence?. *Anaesthesia and intensive care*, *41*(4), 458–462. https://doi.org/10.1177/0310057X1304100404** |
| **213.**Genetic Obesity and the Risk of Atrial Fibrillation: Causal Estimates from Mendelian Randomization | **Chatterjee, N. A., Giulianini, F., Geelhoed, B., Lunetta, K. L., Misialek, J. R., Niemeijer, M. N., Rienstra, M., Rose, L. M., Smith, A. V., Arking, D. E., Ellinor, P. T., Heeringa, J., Lin, H., Lubitz, S. A., Soliman, E. Z., Verweij, N., Alonso, A., Benjamin, E. J., Gudnason, V., Stricker, B. H. C., … Albert, C. M. (2017). Genetic Obesity and the Risk of Atrial Fibrillation: Causal Estimates from Mendelian Randomization. *Circulation*, *135*(8), 741–754. https://doi.org/10.1161/CIRCULATIONAHA.116.024921** |
| **214.**Effects of milk replacer feeding rates on growth performance of Holstein dairy calves to 4 months of age, evaluated via a meta-analytical approach | **Hu, W., Hill, T. M., Dennis, T. S., Suarez-Mena, F. X., Aragona, K. M., Quigley, J. D., & Schlotterbeck, R. L. (2020). Effects of milk replacer feeding rates on growth performance of Holstein dairy calves to 4 months of age, evaluated via a meta-analytical approach. *Journal of dairy science*, *103*(3), 2217–2232. https://doi.org/10.3168/jds.2019-17206** |
| **215.**Age and association of kidney measures with mortality and end-stage renal disease | **Hallan, S. I., Matsushita, K., Sang, Y., Mahmoodi, B. K., Black, C., Ishani, A., Kleefstra, N., Naimark, D., Roderick, P., Tonelli, M., Wetzels, J. F., Astor, B. C., Gansevoort, R. T., Levin, A., Wen, C. P., Coresh, J., & Chronic Kidney Disease Prognosis Consortium (2012). Age and association of kidney measures with mortality and end-stage renal disease. *JAMA*, *308*(22), 2349–2360. https://doi.org/10.1001/jama.2012.16817** |
| **216.**Sodium-Glucose Cotransporter-2 Inhibitor for Renal Function Preservation in Patients with Type 2 Diabetes Mellitus: A Korean Diabetes Association and Korean Society of Nephrology Consensus Statement | **Oh, T. J., Moon, J. Y., Hur, K. Y., Ko, S. H., Kim, H. J., Kim, T., Lee, D. W., Moon, M. K., Committee of Clinical Practice Guideline, Korean Diabetes Association, & Committee of the Cooperative Studies, Korean Society of Nephrology (2020). Sodium-Glucose Cotransporter-2 Inhibitor for Renal Function Preservation in Patients with Type 2 Diabetes Mellitus: A Korean Diabetes Association and Korean Society of Nephrology Consensus Statement. *Diabetes & metabolism journal*, *44*(4), 489–497. https://doi.org/10.4093/dmj.2020.0172** |
| **217.**Interaction between genes and macronutrient intake on the risk of developing type 2 diabetes: systematic review and findings from European Prospective Investigation into Cancer (EPIC)-InterAct | **Li, S. X., Imamura, F., Ye, Z., Schulze, M. B., Zheng, J., Ardanaz, E., Arriola, L., Boeing, H., Dow, C., Fagherazzi, G., Franks, P. W., Agudo, A., Grioni, S., Kaaks, R., Katzke, V. A., Key, T. J., Khaw, K. T., Mancini, F. R., Navarro, C., Nilsson, P. M., … Wareham, N. J. (2017). Interaction between genes and macronutrient intake on the risk of developing type 2 diabetes: systematic review and findings from European Prospective Investigation into Cancer (EPIC)-InterAct. *The American journal of clinical nutrition*, *106*(1), 263–275. https://doi.org/10.3945/ajcn.116.150094** |
| **218.**Milk Fat Globule Membrane Supplementation in Children: Systematic Review with Meta-Analysis | **Ambrożej, D., Dumycz, K., Dziechciarz, P., & Ruszczyński, M. (2021). Milk Fat Globule Membrane Supplementation in Children: Systematic Review with Meta-Analysis. *Nutrients*, *13*(3), 714. https://doi.org/10.3390/nu13030714** |
| **219.**Trends and dietary determinants of overweight and obesity in a multiethnic population | **Maskarinec, G., Takata, Y., Pagano, I., Carlin, L., Goodman, M. T., Le Marchand, L., Nomura, A. M., Wilkens, L. R., & Kolonel, L. N. (2006). Trends and dietary determinants of overweight and obesity in a multiethnic population. *Obesity (Silver Spring, Md.)*, *14*(4), 717–726. https://doi.org/10.1038/oby.2006.82** |
| **220.**Genome Wide Association Study Identifies the HMGCS2 Locus to be Associated With Chlorthalidone Induced Glucose Increase in Hypertensive Patients | **Singh, S., McDonough, C. W., Gong, Y., Alghamdi, W. A., Arwood, M. J., Bargal, S. A., Dumeny, L., Li, W. Y., Mehanna, M., Stockard, B., Yang, G., de Oliveira, F. A., Fredette, N. C., Shahin, M. H., Bailey, K. R., Beitelshees, A. L., Boerwinkle, E., Chapman, A. B., Gums, J. G., Turner, S. T., … Johnson, J. A. (2018). Genome Wide Association Study Identifies the *HMGCS2* Locus to be Associated With Chlorthalidone Induced Glucose Increase in Hypertensive Patients. *Journal of the American Heart Association*, *7*(6), e007339. https://doi.org/10.1161/JAHA.117.007339** |
| **221.**Genome-wide association study to identify common variants associated with brachial circumference: a meta-analysis of 14 cohorts | **Boraska, V., Day-Williams, A., Franklin, C. S., Elliott, K. S., Panoutsopoulou, K., Tachmazidou, I., Albrecht, E., Bandinelli, S., Beilin, L. J., Bochud, M., Cadby, G., Ernst, F., Evans, D. M., Hayward, C., Hicks, A. A., Huffman, J., Huth, C., James, A. L., Klopp, N., Kolcic, I., … Zeggini, E. (2012). Genome-wide association study to identify common variants associated with brachial circumference: a meta-analysis of 14 cohorts. *PloS one*, *7*(3), e31369. https://doi.org/10.1371/journal.pone.0031369** |
| **222.**The effect of disease associated point mutations on 5β-reductase (AKR1D1) enzyme function | **Mindnich, R., Drury, J. E., & Penning, T. M. (2011). The effect of disease associated point mutations on 5β-reductase (AKR1D1) enzyme function. *Chemico-biological interactions*, *191*(1-3), 250–254. https://doi.org/10.1016/j.cbi.2010.12.020** |
| **223.**Brief motivational interventions for college student drinking may not be as powerful as we think: an individual participant-level data meta-analysis | **Huh, D., Mun, E. Y., Larimer, M. E., White, H. R., Ray, A. E., Rhew, I. C., Kim, S. Y., Jiao, Y., & Atkins, D. C. (2015). Brief motivational interventions for college student drinking may not be as powerful as we think: an individual participant-level data meta-analysis. *Alcoholism, clinical and experimental research*, *39*(5), 919–931. https://doi.org/10.1111/acer.12714** |
| **224.**Physical activity attenuates the influence of FTO variants on obesity risk: a meta-analysis of 218,166 adults and 19,268 children | **Kilpeläinen, T. O., Qi, L., Brage, S., Sharp, S. J., Sonestedt, E., Demerath, E., Ahmad, T., Mora, S., Kaakinen, M., Sandholt, C. H., Holzapfel, C., Autenrieth, C. S., Hyppönen, E., Cauchi, S., He, M., Kutalik, Z., Kumari, M., Stančáková, A., Meidtner, K., Balkau, B., … Loos, R. J. (2011). Physical activity attenuates the influence of FTO variants on obesity risk: a meta-analysis of 218,166 adults and 19,268 children. *PLoS medicine*, *8*(11), e1001116. https://doi.org/10.1371/journal.pmed.1001116** |
| **225.**Efficacy and Safety of Continuous Subcutaneous Insulin Infusion vs. Multiple Daily Injections on Type 1 Diabetes Children: A Meta-Analysis of Randomized Control Trials | **Qin, Y., Yang, L. H., Huang, X. L., Chen, X. H., & Yao, H. (2018). Efficacy and Safety of Continuous Subcutaneous Insulin Infusion vs. Multiple Daily Injections on Type 1 Diabetes Children: A Meta-Analysis of Randomized Control Trials. *Journal of clinical research in pediatric endocrinology*, *10*(4), 316–323. https://doi.org/10.4274/jcrpe.0053** |
| **226.**Separate and combined associations of body-mass index and abdominal adiposity with cardiovascular disease: collaborative analysis of 58 prospective studies | **Emerging Risk Factors Collaboration, Wormser, D., Kaptoge, S., Di Angelantonio, E., Wood, A. M., Pennells, L., Thompson, A., Sarwar, N., Kizer, J. R., Lawlor, D. A., Nordestgaard, B. G., Ridker, P., Salomaa, V., Stevens, J., Woodward, M., Sattar, N., Collins, R., Thompson, S. G., Whitlock, G., & Danesh, J. (2011). Separate and combined associations of body-mass index and abdominal adiposity with cardiovascular disease: collaborative analysis of 58 prospective studies. *Lancet (London, England)*, *377*(9771), 1085–1095. https://doi.org/10.1016/S0140-6736(11)60105-0** |
| **227.**Are there secondary effects on marijuana use from brief alcohol interventions for college students? | **White, H. R., Jiao, Y., Ray, A. E., Huh, D., Atkins, D. C., Larimer, M. E., Fromme, K., Corbin, W. R., Baer, J. S., LaBrie, J. W., & Mun, E. Y. (2015). Are there secondary effects on marijuana use from brief alcohol interventions for college students?. *Journal of studies on alcohol and drugs*, *76*(3), 367–377. https://doi.org/10.15288/jsad.2015.76.367** |
| **228.**Lower estimated GFR and higher albuminuria are associated with adverse kidney outcomes. A collaborative meta-analysis of general and high-risk population cohorts | **Gansevoort, R. T., Matsushita, K., van der Velde, M., Astor, B. C., Woodward, M., Levey, A. S., de Jong, P. E., Coresh, J., & Chronic Kidney Disease Prognosis Consortium (2011). Lower estimated GFR and higher albuminuria are associated with adverse kidney outcomes. A collaborative meta-analysis of general and high-risk population cohorts. *Kidney international*, *80*(1), 93–104. https://doi.org/10.1038/ki.2010.531** |
| **229.**Rates of nausea and vomiting in pregnancy and dietary characteristics across populations | **Pepper, G. V., & Craig Roberts, S. (2006). Rates of nausea and vomiting in pregnancy and dietary characteristics across populations. *Proceedings. Biological sciences*, *273*(1601), 2675–2679. https://doi.org/10.1098/rspb.2006.3633** |
| **230.**FTO genotype is associated with phenotypic variability of body mass index | **Yang, J., Loos, R. J., Powell, J. E., Medland, S. E., Speliotes, E. K., Chasman, D. I., Rose, L. M., Thorleifsson, G., Steinthorsdottir, V., Mägi, R., Waite, L., Smith, A. V., Yerges-Armstrong, L. M., Monda, K. L., Hadley, D., Mahajan, A., Li, G., Kapur, K., Vitart, V., Huffman, J. E., … Visscher, P. M. (2012). FTO genotype is associated with phenotypic variability of body mass index. *Nature*, *490*(7419), 267–272. https://doi.org/10.1038/nature11401** |
| **231.**Genetic variant in fat mass and obesity-associated gene associated with type 2 diabetes risk in Han Chinese | **Qian, Y., Liu, S., Lu, F., Li, H., Dong, M., Lin, Y., Du, J., Lin, Y., Gong, J., Jin, G., Dai, J., Hu, Z., & Shen, H. (2013). Genetic variant in fat mass and obesity-associated gene associated with type 2 diabetes risk in Han Chinese. *BMC genetics*, *14*, 86. https://doi.org/10.1186/1471-2156-14-86** |
| **232.**Ketorolac therapy for the prevention of acute pseudophakic cystoid macular edema: a systematic review | **Yilmaz, T., Cordero-Coma, M., & Gallagher, M. J. (2012). Ketorolac therapy for the prevention of acute pseudophakic cystoid macular edema: a systematic review. *Eye (London, England)*, *26*(2), 252–258. https://doi.org/10.1038/eye.2011.296** |
| **233.**New insight into human sweet taste: a genome-wide association study of the perception and intake of sweet substances | **Hwang, L. D., Lin, C., Gharahkhani, P., Cuellar-Partida, G., Ong, J. S., An, J., Gordon, S. D., Zhu, G., MacGregor, S., Lawlor, D. A., Breslin, P. A. S., Wright, M. J., Martin, N. G., & Reed, D. R. (2019). New insight into human sweet taste: a genome-wide association study of the perception and intake of sweet substances. *The American journal of clinical nutrition*, *109*(6), 1724–1737. https://doi.org/10.1093/ajcn/nqz043** |
| **234.**Genome-wide physical activity interactions in adiposity - A meta-analysis of 200,452 adults | **Graff, M., Scott, R. A., Justice, A. E., Young, K. L., Feitosa, M. F., Barata, L., Winkler, T. W., Chu, A. Y., Mahajan, A., Hadley, D., Xue, L., Workalemahu, T., Heard-Costa, N. L., den Hoed, M., Ahluwalia, T. S., Qi, Q., Ngwa, J. S., Renström, F., Quaye, L., Eicher, J. D., … Kilpeläinen, T. O. (2017). Genome-wide physical activity interactions in adiposity - A meta-analysis of 200,452 adults. *PLoS genetics*, *13*(4), e1006528. https://doi.org/10.1371/journal.pgen.1006528** |
| **235.**Genome-wide association for abdominal subcutaneous and visceral adipose reveals a novel locus for visceral fat in women | **Fox, C. S., Liu, Y., White, C. C., Feitosa, M., Smith, A. V., Heard-Costa, N., Lohman, K., GIANT Consortium, MAGIC Consortium, GLGC Consortium, Johnson, A. D., Foster, M. C., Greenawalt, D. M., Griffin, P., Ding, J., Newman, A. B., Tylavsky, F., Miljkovic, I., Kritchevsky, S. B., Launer, L., … Borecki, I. B. (2012). Genome-wide association for abdominal subcutaneous and visceral adipose reveals a novel locus for visceral fat in women. *PLoS genetics*, *8*(5), e1002695. https://doi.org/10.1371/journal.pgen.1002695** |
| **236.**Metabolic characteristics of human subcutaneous abdominal adipose tissue after overnight fast | **Frayn, K. N., & Humphreys, S. M. (2012). Metabolic characteristics of human subcutaneous abdominal adipose tissue after overnight fast. *American journal of physiology. Endocrinology and metabolism*, *302*(4), E468–E475. https://doi.org/10.1152/ajpendo.00527.2011** |
| **237.**Topical NSAIDs for acute pain: a meta-analysis | **Mason, L., Moore, R. A., Edwards, J. E., Derry, S., & McQuay, H. J. (2004). Topical NSAIDs for acute pain: a meta-analysis. *BMC family practice*, *5*, 10. https://doi.org/10.1186/1471-2296-5-10** |
| **238.**[Systematic review with meta-analysis: Subcutaneous insulin glargine coadministration for diabetic ketoacidosis] | **Andrade-Castellanos, C. A., & Colunga-Lozano, L. E. (2016). Revisión sistemática con metaanálisis. Coadministración de insulina glargina en el manejo de la cetoacidosis diabética (CAD) [Systematic review with meta-analysis: Subcutaneous insulin glargine coadministration for diabetic ketoacidosis]. *Gaceta medica de Mexico*, *152*(6), 761–769.** |
| **239.**Is the adiposity-associated FTO gene variant related to all-cause mortality independent of adiposity? Meta-analysis of data from 169,551 Caucasian adults | **Zimmermann, E., Ängquist, L. H., Mirza, S. S., Zhao, J. H., Chasman, D. I., Fischer, K., Qi, Q., Smith, A. V., Thinggaard, M., Jarczok, M. N., Nalls, M. A., Trompet, S., Timpson, N. J., Schmidt, B., Jackson, A. U., Lyytikäinen, L. P., Verweij, N., Mueller-Nurasyid, M., Vikström, M., Marques-Vidal, P., … Sørensen, T. I. A. (2015). Is the adiposity-associated FTO gene variant related to all-cause mortality independent of adiposity? Meta-analysis of data from 169,551 Caucasian adults. *Obesity reviews : an official journal of the International Association for the Study of Obesity*, *16*(4), 327–340. https://doi.org/10.1111/obr.12263** |
| **240.**Safety of four SGLT2 inhibitors in three chronic diseases: A meta-analysis of large randomized trials of SGLT2 inhibitors | **Qiu, M., Ding, L. L., Zhang, M., & Zhou, H. R. (2021). Safety of four SGLT2 inhibitors in three chronic diseases: A meta-analysis of large randomized trials of SGLT2 inhibitors. *Diabetes & vascular disease research*, *18*(2), 14791641211011016. https://doi.org/10.1177/14791641211011016** |
| **241.**Using genetics to test the causal relationship of total adiposity and periodontitis: Mendelian randomization analyses in the Gene-Lifestyle Interactions and Dental Endpoints (GLIDE) Consortium | **Shungin, D., Cornelis, M. C., Divaris, K., Holtfreter, B., Shaffer, J. R., Yu, Y. H., Barros, S. P., Beck, J. D., Biffar, R., Boerwinkle, E. A., Crout, R. J., Ganna, A., Hallmans, G., Hindy, G., Hu, F. B., Kraft, P., McNeil, D. W., Melander, O., Moss, K. L., North, K. E., … Franks, P. W. (2015). Using genetics to test the causal relationship of total adiposity and periodontitis: Mendelian randomization analyses in the Gene-Lifestyle Interactions and Dental Endpoints (GLIDE) Consortium. *International journal of epidemiology*, *44*(2), 638–650. https://doi.org/10.1093/ije/dyv075** |
| **242.**Prognostic value of aldo-keto reductase family 1 member B10 (AKR1B10) in digestive system cancers: A meta-analysis | **Liu, R., Zheng, S., Yang, C. Y., Yu, Y., Peng, S., Ge, Q., Lin, Q., Li, Q., Shi, W., & Shao, Y. (2021). Prognostic value of aldo-keto reductase family 1 member B10 (AKR1B10) in digestive system cancers: A meta-analysis. *Medicine*, *100*(14), e25454. https://doi.org/10.1097/MD.0000000000025454** |
| **243.**Association of genetic variation in FTO with risk of obesity and type 2 diabetes with data from 96,551 East and South Asians | **Li, H., Kilpeläinen, T. O., Liu, C., Zhu, J., Liu, Y., Hu, C., Yang, Z., Zhang, W., Bao, W., Cha, S., Wu, Y., Yang, T., Sekine, A., Choi, B. Y., Yajnik, C. S., Zhou, D., Takeuchi, F., Yamamoto, K., Chan, J. C., Mani, K. R., … Loos, R. J. (2012). Association of genetic variation in FTO with risk of obesity and type 2 diabetes with data from 96,551 East and South Asians. *Diabetologia*, *55*(4), 981–995. https://doi.org/10.1007/s00125-011-2370-7** |
| **244.**Genome-wide meta-analysis of muscle weakness identifies 15 susceptibility loci in older men and women | **Jones, G., Trajanoska, K., Santanasto, A. J., Stringa, N., Kuo, C. L., Atkins, J. L., Lewis, J. R., Duong, T., Hong, S., Biggs, M. L., Luan, J., Sarnowski, C., Lunetta, K. L., Tanaka, T., Wojczynski, M. K., Cvejkus, R., Nethander, M., Ghasemi, S., Yang, J., Zillikens, M. C., … Pilling, L. C. (2021). Genome-wide meta-analysis of muscle weakness identifies 15 susceptibility loci in older men and women. *Nature communications*, *12*(1), 654. https://doi.org/10.1038/s41467-021-20918-w** |
| **245.**Variability among nonsteroidal antiinflammatory drugs in risk of upper gastrointestinal bleeding | **Massó González, E. L., Patrignani, P., Tacconelli, S., & García Rodríguez, L. A. (2010). Variability among nonsteroidal antiinflammatory drugs in risk of upper gastrointestinal bleeding. *Arthritis and rheumatism*, *62*(6), 1592–1601. https://doi.org/10.1002/art.27412** |
| **246.**Associations of genetic variants in/near body mass index-associated genes with type 2 diabetes: a systematic meta-analysis | **Xi, B., Takeuchi, F., Meirhaeghe, A., Kato, N., Chambers, J. C., Morris, A. P., Cho, Y. S., Zhang, W., Mohlke, K. L., Kooner, J. S., Shu, X. O., Pan, H., Tai, E. S., Pan, H., Wu, J. Y., Zhou, D., Chandak, G. R., DIAGRAM Consortium, AGEN-T2D Consortium, & SAT2D Consortium (2014). Associations of genetic variants in/near body mass index-associated genes with type 2 diabetes: a systematic meta-analysis. *Clinical endocrinology*, *81*(5), 702–710. https://doi.org/10.1111/cen.12428** |
| **247.**Single-dose ketorolac and pethidine in acute postoperative pain: systematic review with meta-analysis | **Smith, L. A., Carroll, D., Edwards, J. E., Moore, R. A., & McQuay, H. J. (2000). Single-dose ketorolac and pethidine in acute postoperative pain: systematic review with meta-analysis. *British journal of anaesthesia*, *84*(1), 48–58. https://doi.org/10.1093/oxfordjournals.bja.a013381** |
| **248.**[Association of early nutrition deficiency with the risk of bronchopulmonary dysplasia: a Meta analysis] | **Fang, L. Y., Chen, D. M., Han, S. P., Chen, X. H., & Yu, Z. B. (2021). *Zhongguo dang dai er ke za zhi = Chinese journal of contemporary pediatrics*, *23*(4), 390–396. https://doi.org/10.7499/j.issn.1008-8830.2011094** |
| **249.**Adult height and the risk of cause-specific death and vascular morbidity in 1 million people: individual participant meta-analysis | **Emerging Risk Factors Collaboration (2012). Adult height and the risk of cause-specific death and vascular morbidity in 1 million people: individual participant meta-analysis. *International journal of epidemiology*, *41*(5), 1419–1433. https://doi.org/10.1093/ije/dys086** |
| **250.**A variant within the FTO confers susceptibility to diabetic nephropathy in Japanese patients with type 2 diabetes | **Taira, M., Imamura, M., Takahashi, A., Kamatani, Y., Yamauchi, T., Araki, S. I., Tanaka, N., van Zuydam, N. R., Ahlqvist, E., Toyoda, M., Umezono, T., Kawai, K., Imanishi, M., Watada, H., Suzuki, D., Maegawa, H., Babazono, T., Kaku, K., Kawamori, R., SUMMIT Consortium, … Maeda, S. (2018). A variant within the FTO confers susceptibility to diabetic nephropathy in Japanese patients with type 2 diabetes. *PloS one*, *13*(12), e0208654. https://doi.org/10.1371/journal.pone.0208654** |
| **251.**Dose-response relationships between individual nonaspirin nonsteroidal anti-inflammatory drugs (NANSAIDs) and serious upper gastrointestinal bleeding: a meta-analysis based on individual patient data | **Lewis, S. C., Langman, M. J., Laporte, J. R., Matthews, J. N., Rawlins, M. D., & Wiholm, B. E. (2002). Dose-response relationships between individual nonaspirin nonsteroidal anti-inflammatory drugs (NANSAIDs) and serious upper gastrointestinal bleeding: a meta-analysis based on individual patient data. *British journal of clinical pharmacology*, *54*(3), 320–326. https://doi.org/10.1046/j.1365-2125.2002.01636.x** |
| **252.**Antifungal prophylaxis for severely neutropenic chemotherapy recipients: a meta analysis of randomized-controlled clinical trials | **Bow, E. J., Laverdière, M., Lussier, N., Rotstein, C., Cheang, M. S., & Ioannou, S. (2002). Antifungal prophylaxis for severely neutropenic chemotherapy recipients: a meta analysis of randomized-controlled clinical trials. *Cancer*, *94*(12), 3230–3246. https://doi.org/10.1002/cncr.10610** |
| **253.**Treatment of patients with metastatic renal cell cancer: a RAND Appropriateness Panel | **Halbert, R. J., Figlin, R. A., Atkins, M. B., Bernal, M., Hutson, T. E., Uzzo, R. G., Bukowski, R. M., Khan, K. D., Wood, C. G., & Dubois, R. W. (2006). Treatment of patients with metastatic renal cell cancer: a RAND Appropriateness Panel. *Cancer*, *107*(10), 2375–2383. https://doi.org/10.1002/cncr.22260** |
| **254.**Quantitative systematic review of topically applied non-steroidal anti-inflammatory drugs | **Moore, R. A., Tramèr, M. R., Carroll, D., Wiffen, P. J., & McQuay, H. J. (1998). Quantitative systematic review of topically applied non-steroidal anti-inflammatory drugs. *BMJ (Clinical research ed.)*, *316*(7128), 333–338. https://doi.org/10.1136/bmj.316.7128.333** |
| **255.**Sodium-glucose cotransporter 2 inhibitors as an add-on therapy to insulin for type 1 diabetes mellitus: Meta-analysis of randomized controlled trials | **Rao, L., Ren, C., Luo, S., Huang, C., & Li, X. (2021). Sodium-glucose cotransporter 2 inhibitors as an add-on therapy to insulin for type 1 diabetes mellitus: Meta-analysis of randomized controlled trials. *Acta diabetologica*, *58*(7), 869–880. https://doi.org/10.1007/s00592-021-01686-x** |
| **256.**Variability in risk of gastrointestinal complications with individual non-steroidal anti-inflammatory drugs: results of a collaborative meta-analysis | **Henry, D., Lim, L. L., Garcia Rodriguez, L. A., Perez Gutthann, S., Carson, J. L., Griffin, M., Savage, R., Logan, R., Moride, Y., Hawkey, C., Hill, S., & Fries, J. T. (1996). Variability in risk of gastrointestinal complications with individual non-steroidal anti-inflammatory drugs: results of a collaborative meta-analysis. *BMJ (Clinical research ed.)*, *312*(7046), 1563–1566. https://doi.org/10.1136/bmj.312.7046.1563** |
| **257.**Advancing methods for reliably assessing motivational interviewing fidelity using the motivational interviewing skills code | **Lord, S. P., Can, D., Yi, M., Marin, R., Dunn, C. W., Imel, Z. E., Georgiou, P., Narayanan, S., Steyvers, M., & Atkins, D. C. (2015). Advancing methods for reliably assessing motivational interviewing fidelity using the motivational interviewing skills code. *Journal of substance abuse treatment*, *49*, 50–57. https://doi.org/10.1016/j.jsat.2014.08.005** |
| **258.**Telithromycin in the treatment of community-acquired pneumonia: a pooled analysis | **Hagberg, L., Carbon, C., van Rensburg, D. J., Fogarty, C., Dunbar, L., & Pullman, J. (2003). Telithromycin in the treatment of community-acquired pneumonia: a pooled analysis. *Respiratory medicine*, *97*(6), 625–633. https://doi.org/10.1053/rmed.2003.1492** |
| **259.**Population pharmacokinetic modelling of darifenacin and its hydroxylated metabolite using pooled data, incorporating saturable first-pass metabolism, CYP2D6 genotype and formulation-dependent bioavailability | **Kerbusch, T., Wählby, U., Milligan, P. A., & Karlsson, M. O. (2003). Population pharmacokinetic modelling of darifenacin and its hydroxylated metabolite using pooled data, incorporating saturable first-pass metabolism, CYP2D6 genotype and formulation-dependent bioavailability. *British journal of clinical pharmacology*, *56*(6), 639–652. https://doi.org/10.1046/j.1365-2125.2003.01967.x** |
| **260.**Towards evidence-based emergency medicine: best BETs from the Manchester Royal Infirmary. Venous blood gas in adult patients with diabetic ketoacidosis | **Hassan, Z., Subramonyam, D. M., & Thakore, S. (2003). Towards evidence based emergency medicine: best BETs from the Manchester Royal Infirmary. Venous blood gas in adult patients with diabetic ketoacidosis. *Emergency medicine journal : EMJ*, *20*(4), 363–364. https://doi.org/10.1136/emj.20.4.363** |
| **261.**The efficacy of nonopioid analgesics for postoperative dental pain: a meta-analysis | **Ahmad, N., Grad, H. A., Haas, D. A., Aronson, K. J., Jokovic, A., & Locker, D. (1997). The efficacy of nonopioid analgesics for postoperative dental pain: a meta-analysis. *Anesthesia progress*, *44*(4), 119–126.** |
| **262.**Diet-Related Alterations of Gut Bile Salt Hydrolases Determined Using a Metagenomic Analysis of the Human Microbiome | **Jia, B., Park, D., Chun, B. H., Hahn, Y., & Jeon, C. O. (2021). Diet-Related Alterations of Gut Bile Salt Hydrolases Determined Using a Metagenomic Analysis of the Human Microbiome. *International journal of molecular sciences*, *22*(7), 3652. https://doi.org/10.3390/ijms22073652** |
| **263.**Effects of smoking on the genetic risk of obesity: the population architecture using genomics and epidemiology study | **Fesinmeyer, M. D., North, K. E., Lim, U., Bůžková, P., Crawford, D. C., Haessler, J., Gross, M. D., Fowke, J. H., Goodloe, R., Love, S. A., Graff, M., Carlson, C. S., Kuller, L. H., Matise, T. C., Hong, C. P., Henderson, B. E., Allen, M., Rohde, R. R., Mayo, P., Schnetz-Boutaud, N., … Peters, U. (2013). Effects of smoking on the genetic risk of obesity: the population architecture using genomics and epidemiology study. *BMC medical genetics*, *14*, 6. https://doi.org/10.1186/1471-2350-14-6** |
| **264.**The effects of nonsteroidal anti-inflammatory drugs (NSAIDs) on postoperative renal function: a meta-analysis | **Lee, A., Cooper, M. G., Craig, J. C., Knight, J. F., & Keneally, J. P. (1999). The effects of nonsteroidal anti-inflammatory drugs (NSAIDs) on postoperative renal function: a meta-analysis. *Anaesthesia and intensive care*, *27*(6), 574–580. https://doi.org/10.1177/0310057X9902700603** |
| **265.**Type 1 Diabetes Mellitus and Cognitive Impairments: A Systematic Review | **Li, W., Huang, E., & Gao, S. (2017). Type 1 Diabetes Mellitus and Cognitive Impairments: A Systematic Review. *Journal of Alzheimer's disease : JAD*, *57*(1), 29–36. https://doi.org/10.3233/JAD-161250** |
| **266.**Associations of Diet and Physical Activity with Risk for Gestational Diabetes Mellitus: A Systematic Review and Meta-Analysis | **Mijatovic-Vukas, J., Capling, L., Cheng, S., Stamatakis, E., Louie, J., Cheung, N. W., Markovic, T., Ross, G., Senior, A., Brand-Miller, J. C., & Flood, V. M. (2018). Associations of Diet and Physical Activity with Risk for Gestational Diabetes Mellitus: A Systematic Review and Meta-Analysis. *Nutrients*, *10*(6), 698. https://doi.org/10.3390/nu10060698** |
| **267.**Dietary carbohydrate intake and mortality: a prospective cohort study and meta-analysis | **Seidelmann, S. B., Claggett, B., Cheng, S., Henglin, M., Shah, A., Steffen, L. M., Folsom, A. R., Rimm, E. B., Willett, W. C., & Solomon, S. D. (2018). Dietary carbohydrate intake and mortality: a prospective cohort study and meta-analysis. *The Lancet. Public health*, *3*(9), e419–e428. https://doi.org/10.1016/S2468-2667(18)30135-X** |
